# Tuning Li^+^ and Na^+^ Functionality in Renewable Carbon Electroactive Material Through Site‐Specific Nanostructural Disorder

**DOI:** 10.1002/advs.202523383

**Published:** 2026-01-31

**Authors:** Montajar Sarkar, Rumana Hossain, Jian Peng, Dilan Kumara Thilakarathna, Yu Wang, Yin Yao, Neeraj Sharma, Veena Sahajwalla

**Affiliations:** ^1^ Centre For Sustainable Materials Research and Technology SMaRT@UNSW School of Materials Science and Engineering UNSW Sydney Sydney New South Wales Australia; ^2^ School of Chemistry UNSW Sydney Sydney New South Wales Australia; ^3^ Solid State & Elemental Analysis Unit (SSEAU) X‐ray Diffraction Laboratory Mark Wainwright Analytical Centre UNSW Sydney Sydney New South Wales Australia; ^4^ Electron Microscope Unit (EMU) Mark Wainwright Analytical Centre UNSW Sydney Sydney New South Wales Australia

**Keywords:** carbons, lithium ion battery, microstructure, sodium ion battery

## Abstract

Understanding the hybrid structural characteristics of carbon remains a significant challenge, hindering the development of clear design principles for optimizing electrode materials in energy storage systems—particularly in lithium‐ion (Li‐ion) and sodium‐ion (Na‐ion) batteries. Traditionally, the localized structural disorder and intrinsic porosity of carbon have been regarded as the primary contributors to its high storage capacity in Na‐ion batteries. However, our investigation reveals that the hybrid structural features of carbon play a more dominant role in enhancing energy storage performance in Li‐ion systems compared to Na‐ion counterparts. Through comprehensive materials characterization and electrochemical analysis, we establish a direct correlation between the local microstructural attributes of carbon and its ion storage kinetics which elucidate the distinct ion‐storage mechanisms that differentiate Li‐ion from Na‐ion systems. This study reinforces our findings that configurational defects are crucial for achieving high initial storage capacity in carbon crafted with mixed‐phase structures, but the in‐plane size of nano‐layered microdomains plays a key role in ensuring long‐term stable storage capacity in rechargeable batteries.

## Graphical abstract

This study presents a selective thermal transformation of polycarbonate into hybrid carbon materials. The structured carbon enhances electrochemical performance, particularly in lithium‐ion systems. Investigations reveal improved bimetallic ion diffusivity through the hybrid microstructure, contributing to excellent charge kinetics. Compared to sodium‐ion systems, lithium‐ion cells exhibit significantly better energy storage and rate capability, highlighting the potential of engineered carbon architectures for high‐performance energy applications.

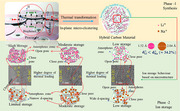

## 1 Introduction

The dumping of e‐waste has become an increasingly serious global issue, largely due to the short service life of modern electronic devices. To achieve sustainable development, responsible consumption and production must address the growing challenges associated with e‐waste by adopting sustainable strategies for its management. Efficient recycling and proper handling of e‐waste are crucial, as improper disposal continues to contribute significantly to landfill growth. Currently, only about 20% of e‐waste is recycled, while approximately 80% remains uncollected due to high processing costs and a lack of adequate infrastructure [1]. Among various forms of e‐waste, compact discs (CDs) present a notable concern. The end‐of‐life scenario for CD waste remains ambiguous. As society increasingly transitions to digital platforms and embraces circular economy principles, questions arise about the ultimate fate of these CDs. Improper disposal of CDs is particularly problematic, as the polycarbonate [2] used in their production can depolymerize into bisphenol A [3, 4], a compound associated with environmental and health risks. Therefore, exploring efficient recycling strategies for CD waste is essential. Continued research into the recovery and transformation of such materials can play a critical role in promoting sustainable waste management practices and advancing the circular economy [5, 6].

Graphite has been the dominant anode material since the commercialization of lithium‐ion batteries (LIBs). For sustainable post‐lithium technologies, sodium‐ion batteries (NIBs) have emerged as a promising alternative [7]. In the search for suitable anode materials for NIBs, carbon has been considered one of the most viable candidates. However, the commonly used graphite cannot be directly applied in NIBs. Although graphite performs satisfactorily as an anode in LIBs, it fails to deliver acceptable electrochemical performance in NIBs—primarily due to its microstructural morphology. As a result, other forms of carbon, particularly hard carbon (e.g., biochar) [8, 9, 10, 11], have been extensively investigated for use in sodium‐ion batteries. For NIBs, the anodic behavior of a material must exhibit a lower sodium‐ion insertion and extraction potential than that of metallic sodium. While highly ordered graphitic carbon meets this criterion effectively for LIBs, they fall short in delivering comparable performance for sodium‐ion systems. In contrast, disordered carbonaceous materials present a promising alternative for NIB anodes. These materials are characterized by a highly disordered structure with nanoscale porosity, formed by stacked and twisted graphene sheets. This unique architecture facilitates efficient sodium‐ion storage during electrochemical cycling, making them well‐suited for use as anode materials in sodium‐ion batteries [11].

Despite graphite's widespread use in LIBs, there are two major limitations: (i) Critical mineral graphite is classified as a material with supply risk [5, 12], and (ii) It is not an effective anode material for NIBs [13]. Therefore, identifying an alternative resource that can serve as a feedstock for producing carbon materials suitable for both LIB and NIB anodes presents a compelling opportunity. Such a solution would reduce dependence on graphite and offer a common anode material for both battery technologies—effectively addressing two challenges with one solution, a true win‐win scenario. To address the aforementioned challenges, we synthesized carbon from waste CDs for application in lithium‐ion (LIBs) and sodium‐ion batteries (NIBs). The resulting carbon microstructure comprises of ordered and disordered carbon phases. Given that even slight variations in these microstructural characteristics can significantly impact electrochemical performance due to differing ion‐storage behaviors, it is essential to thoroughly investigate these features. Such analysis is critical to uncovering the interdependence between material microstructure and performance.

To advance the development high performance energy material for battery technologies, it is essential to explore the applicability of the same electrode materials across different battery systems. In this study, a series of carbon materials with tailored hybrid microstructures were synthesized from waste compact discs (CDs), composed primarily of polycarbonate, and evaluated as electrode materials in both lithium‐ion (LIB) and sodium‐ion batteries (NIB). The synthesis was conducted at temperatures ranging from 1200°C to 1600°C to induce varied microstructural features. Electrochemical performance and ion‐storage behavior were systematically compared between the two systems to elucidate the influence of microstructural characteristics on energy storage capacity and kinetics. Among the samples, the carbon material synthesized at 1600°C demonstrated the highest performance in the LIB system, marking it as the most promising candidate for carbon‐based energy storage. In summary, the hybrid carbon matrix derived from waste CDs exhibited excellent electrochemical behavior in lithium‐ion batteries compared to sodium‐ion batteries, highlighting the critical role of microstructural tuning in optimizing carbon materials for next‐generation energy storage material.

## 2 Results and Discussion

### 2.1 Microstructural and Morphological Progression

**FIGURE 1:**
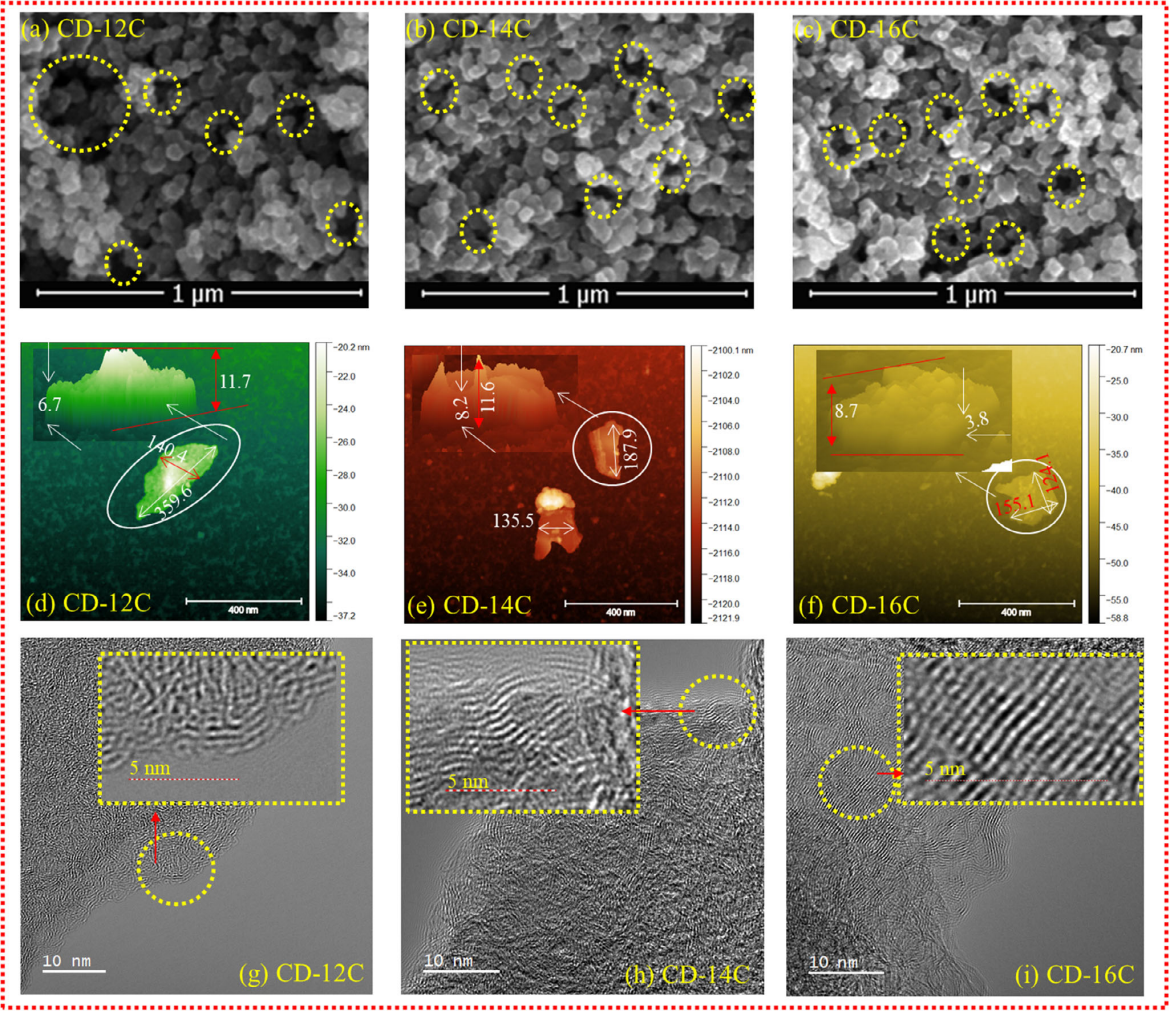
Characterization of carbon samples CD‐12C, CD‐14C, and CD‐16C. (a–c) Scanning electron microscopy (SEM) images outlining the surface morphology of CD‐12C, CD‐14C, and CD‐16C, respectively. (d–f) Atomic force microscopy (AFM) images of the samples, presented in both 2D and 3D views, highlighting surface topography and nanoscale features. (g–i) High‐resolution transmission electron microscopy (HR‐TEM) images revealing the presence of ordered domains and short‐range graphene layers, along with visible interlayer d‐spacing.

The carbon studied here was synthesized through the single‐step selective thermal transformation (SSTT) of waste CDs. The feedstock was carbonized at three selective temperatures of 1200°C, 1400°C, 1600°C for 1 h, yielding the samples CD‐12C, CD‐14C, and CD‐16C, respectively. Selective thermal treatment is a single‐step method to tune the surface chemistry and material properties of the carbon [14, 15]. Scanning electron microscopy (SEM) images of the carbon samples (Figure 1a–c) reveal minimal morphological contrast. As depicted in Figure 1a–c, the carbon particles appear agglomerated, large, and nearly spherical. The agglomeration of nanocarbon structures results in the development of large cavities within the bulk particles, as distinctly indicated by the dotted yellow circles in the SEM images. The spherical morphology of the carbon produced via SSTT is particularly advantageous, as it facilitates uniform electrochemical progression within the electrode during electrochemical kinetics [16]. Figure 1d–e presents atomic force microscopy (AFM) images of individual carbon particles, alongside SEM images. These images reveal that the physical morphology of the carbon particles is consistent across both imaging techniques. The microstructure of the carbons was further analyzed at the nano level using high‐resolution transmission electron microscopy (HR‐TEM). The HR‐TEM images of carbon samples (CD‐12C, CD‐14C, and CD‐16C) are presented in Figure 1a–c, respectively. These high‐resolution images clearly reveal that the carbon samples consist of ordered and disordered microstructures, with short‐range ordered domains embedded within a hybrid carbon matrix. Notably, as the carbonization temperature increases, the size of the ordered domains becomes larger. Figure S1 illustrates the d‐spacing of CD‐12C, CD‐14C, and CD‐16C measured at various locations, along with corresponding histograms (Figure S1d–f) depicting the distribution of d‐spacing values across multiple measurement points. The average interlayer spacings of CD‐12C, CD‐14C, and CD‐16C were measured to be 0.402, 0.373, and 0.36, respectively, indicating a gradual structural improvement with increasing carbonization temperature. Relatively large interlayer spacings are considered to be beneficial for ion intercalation and deintercalation during the charging and discharging cycles of the cells [17].

### 2.2 Micro‐Ordering in Carbon Derived From Thermal Annealing

**FIGURE 2:**
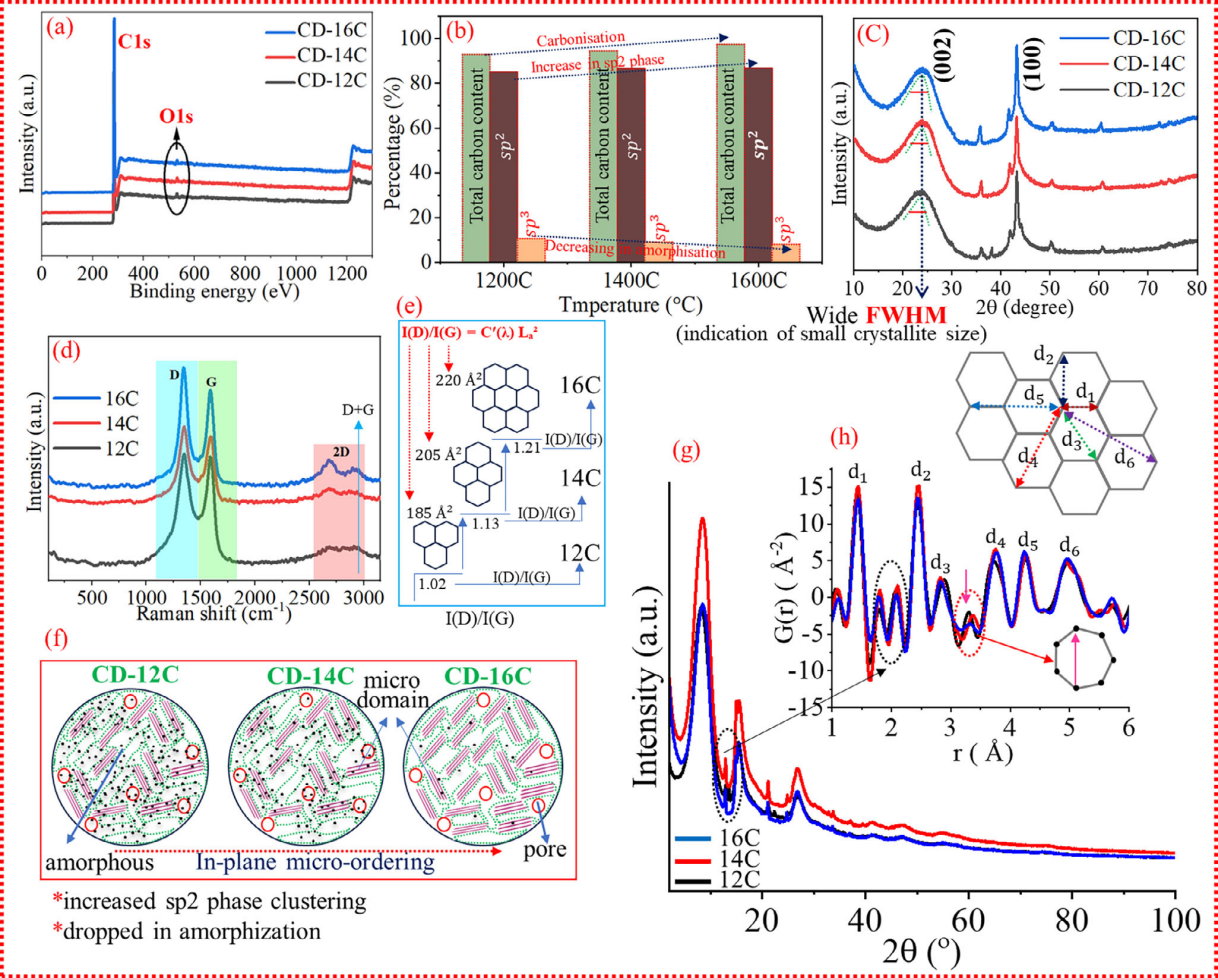
(a) XPS survey spectrum of carbon, highlighting the dominance of C1s and O1s signals. (b) Distribution of total carbon content, showing that the sp^2^‐hybridized phase is predominant, while amorphization decreases with increasing temperature. (c) XRD patterns of the carbon samples, exhibiting a broad (002) reflection indicative of a disordered nature. (d) Raman spectra of the carbon materials. (e) Calculated ordered domain size derived from the Raman D‐to‐G band intensity ratio. (f) Evolution of in‐plane microstructural ordering with increasing temperature. (g) X‐ray diffractogram and (h) corresponding pair distribution function (PDF) analysis, illustrating structural progression in the carbon matrix.

At the initial stage after synthesis, the carbon was analyzed using X‐ray photoelectron spectroscopy (XPS) to investigate its surface chemistry. The survey spectra obtained from XPS is presented in Figure 2a. The elemental composition derived from the analysis, along with the core‐level XPS spectra of C 1s and O 1s (Figures S2–S4), reveals that as the temperature increases, the oxygen content decreases (Tables S1–S3), leading to a reduction in surface oxygen‐containing groups (C─O). The CHNS‐O and X‐ray fluorescence (XRF) analyses of the carbon materials are summarized in Tables S4 and S5. These findings are further corroborated by XPS analysis, which indicates that higher temperatures lead to the loss of oxygen [18, 19] and hydrogen [20]. This suggests that as the temperature increases, oxygen‐ and hydrogen‐containing functional groups, such as hydroxyl groups, gradually decrease [21]. A decrease in the content of surface functionalities is beneficial for achieving high coulombic efficiency during the initial cycling stage. This is because functional groups are considered detrimental to the initial coulombic efficiency (ICE), as they can trigger undesired irreversible chemical reactions [22]. These reactions lead to unwanted electrolyte consumption and permanent ion trapping within the functional groups. The XRD patterns of the carbons are shown in Figure 2c. Two distinct broad diffraction peaks are observed at 2θ ≈ 23° and 43°, corresponding to the (200) and (100) graphitic planes, respectively [23]. The low intensities of these peaks indicate the amorphous nature of the carbons [24, 25]. With an increase in the annealing temperature, the 002 reflection in the X‐ray diffractogram shifts slightly toward a higher 2θ (°) value, accompanied by a decrease in the full width at half maximum (FWHM). To further assess the degree of nano‐crystallinity from in‐plane micro‐ordering of phases during thermal treatment and microstructural defect concentration, Raman spectra were employed. The sharp Raman modes observed around 1580 cm^−1^ (G peak) and 1350 cm^−1^ (D peak) are typically assigned to zone‐center phonons of E2g symmetry and K‐point phonons of A1g symmetry, respectively (Figure 2d) [26]. The measured I(D)/I(G) ratios for CD‐12C, CD‐14C, and CD‐16C are 1.02, 1.13, and 1.21, respectively. The Gaussian fitting curves of Raman spectra are displayed in Figure S5. This intensity ratio of the D‐band to the G‐band, I(D)/I(G), is commonly used to estimate the degree of structural disorder in carbon materials [27]. However, the classical interpretation of the I(D)/I(G) ratio is particularly valid for graphite or micro/nanocrystalline graphite. Applying the same interpretation to hybrid carbon structures—where ordered and disordered carbon coexist at the nano scale—can be misleading. In graphitic materials, the I(D)/I(G) ratio increases with increasing structural disorder (e.g., bond‐angle/bond‐length disorder and hybridization effects), as described by the Tuinstra and Koenig (TK) equation:

(1) $$I\left(D\right)/I\left(G\right)=C\left(\lambda \right)/L_{a}$$

where *L_a_* is the in‐plane crystallite size or correlation length, and *C(λ)* is a constant dependent on the excitation wavelength. In contrast, for amorphous carbon with small *L_a_* values (typically <20 Å), the D‐mode intensity becomes proportional to the probability of sixfold ring formation within the cluster—hence proportional to the cluster area [28]. Therefore, in hybrid carbon structures, the D‐mode can signify increasing order rather than disorder, which is the opposite of what it implies in graphitic carbon. In such cases, the TK equation (Equation 1) is no longer suitable for interpreting the structural ordering. Instead, the following relationship is more appropriate:

(2) $$I\left(D\right)/I\left(G\right)=C^{\prime }\left(\lambda \right)/L_{a}^{2}$$

HR‐TEM analysis (Figure 1g–i) confirms that the carbon studied here has small *L_a_* values (<20 Å) and consists of a mixture of ordered and disordered carbon at the nanoscale. XRD analysis (Figure 2c) reveals the amorphous nature of the carbon, while XPS (Figure 2b) indicates the presence of both sp^2^ and sp^3^ hybridized phases, with sp^2^ being dominant. Thus, in this context, the increasing I(D)/I(G) ratio is interpreted as a measure of structural ordering, and Equation (2) is used to estimate the cluster area (see Figure 2d,e for the projection of band ratio and clustering area as a function of temperature). In this context, the observed microstructural evolution of the carbon can be attributed to two fundamental processes: (i) conversion of sp^3^ to sp^2^ bonding, and (ii) in‐plane micro‐ordering of sp^2^ domains, followed by phase ordering of sp^2^ sites into ring structures. It is now clear that the SSTT process provides an ideal thermal environment that promotes in‐plane micro‐ordering of sp^2^ carbon, ultimately leading to a higher degree of graphitization at the nano scale. The schematic in Figure 2f displays progressive sp^2^ phase ordering and dropped in amorphization with increased in‐plane micro‐ordering. Alongside Raman analysis, the local atomistic structure and the concentration of structural defects were evaluated using pair distribution function (PDF) analysis (Figure 2g,h). The peaks d_1_ to d_6_ (Figure 2h) represent the shortest interatomic distances within the lattice. Distinctive peaks at 1.46 Å (d_1_), 2.44 Å (d_2_), and 2.86 Å (d_3_) indicate the presence of hexagonally bonded sp^2^ carbon domains within the carbon matrix. Additionally, a peak at approximately 3.2 Å suggests the existence of seven‐membered carbon rings [29], whose intensity progressively decreases with increasing in‐plane micro‐ordering. Importantly, these findings demonstrate that Raman analysis alone is insufficient to fully reveal the local microstructure of hybrid carbon materials. Furthermore, the peaks d_4_, d_5_, and d_6_ become more pronounced and sharper from CD‐12C to CD‐16C, indicating enhanced long‐range in‐plane micro‐ordering in the CD‐16C carbon. In addition to the size and long‐range ordering, the concentration of graphitic domains plays a crucial role in defining the properties of these hybrid carbons. XPS analysis (Figure 2b) shows a clear increase in sp^2^ carbon from CD‐12C to CD‐16C, indicating that the proportion of ordered domains grows with temperature. As previously revealed by TEM (Figure 1g–i), carbon consists of randomly oriented, curved graphene nanosheets with a turbostratic structure, making it challenging to draw precise boundaries between individual domains. Nevertheless, approximate domain counts can be obtained from TEM images (Figure S6), where a 225 nm^2^ region was selected and the domains highlighted in yellow. These images show that the number of domains increases as the synthesis temperature rises. Although the hybrid nature of the carbon means that domain concentration may vary across different regions, the overall trend demonstrates that higher temperatures promote an increase in domain number.

### 2.3 Electrochemical Performance of Carbon in Li and Na Systems

**FIGURE 3:**
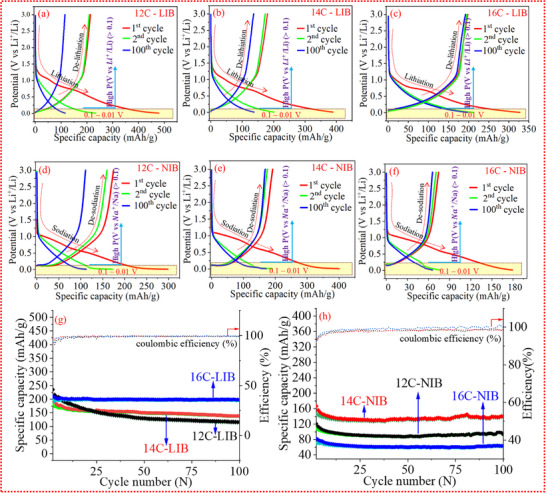
(a–c) Galvanostatic charge–discharge profiles of the anode in the lithium‐ion system at a current density of 50 mA g^−1^ for the first, second, and 100th cycle. (d–f) Galvanostatic charge–discharge profiles of the anode in the sodium‐ion system at a current density of 50 mA g^−1^ for the first, second, and 100th cycle. (g) Cycling performance of the anode in the lithium‐ion battery (LIB) system over 100 cycles at 50 mA g^−1^. (h) Cycling performance of the anode in the sodium‐ion battery (NIB) system over 100 cycles at 50 mA g^−1^.

To delve into the electrochemical kinetics of the materials, Li‐ion and Na‐ion half‐cells were prepared along with the appropriate electrolyte system (see the experimental section). The galvanostatic discharge–charge profiles for the first, second, and 100th cycles at a current density of 50 mA g^−1^, within a potential window of 0.01–3.0 V, are shown in Figure 3a–f. In the case of LIBs, both CD‐12C and CD‐14C initially delivered high capacity, but their capacity gradually faded with increasing cycle number. In contrast, CD‐16C exhibited a different behavior: it delivered a lower initial capacity but showed better capacity retention over cycling. The cyclic performance of the active materials in LIBs is presented in Figure 3g, where the galvanostatic discharge–charge profiles further confirm that CD‐16C exhibits superior electrochemical performance compared to CD‐12C and CD‐14C. This enhanced performance suggests that the microstructural morphology of CD‐16C is more energetically favorable for Li^+^ ion storage. The superior electrochemical behavior of CD‐16C can be attributed to the in‐plane micro‐ordering of carbon structures, which likely results from high‐temperature thermal treatment. When this hybrid carbon structure was tested in Na‐ion batteries alongside Li‐ion batteries to investigate differences in electrochemical kinetics, it was observed that Na‐ion systems exhibit distinct kinetics compared to their Li‐ion counterparts. Among all the carbon samples, CD‐14C delivered excellent electrochemical performance (Figure 3e) however, in direct comparison, this hybrid carbon type performs better in Li‐ion cells (Figure 3g,h). This difference can be attributed not only to the structural and morphological characteristics of the host material but also to the physical properties of the respective metal ions.

**FIGURE 4:**
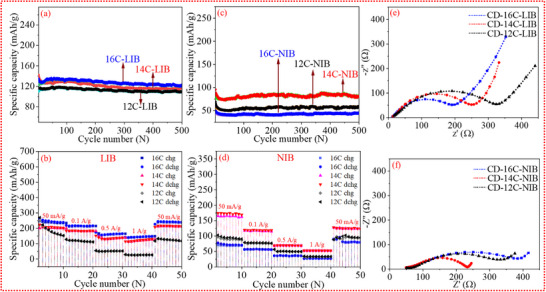
(a) Long‐term cycling performance of the anode in the lithium‐ion battery (LIB) system over 500 cycles at a current density of 500 mA g^−1^. (b) Rate capability of the anode in the LIB system evaluated at various current densities. (c) Long‐term cycling performance of the anode in the sodium‐ion battery (NIB) system over 500 cycles at a current density of 500 mA g^−1^. (d) Rate capability of the anode in the NIB system evaluated at various current densities. (e) Electrochemical impedance spectroscopy (EIS) spectra of the anode in the LIB system. (f) EIS spectra of the anode in the NIB system.

The 16C‐LIB exhibited a lower specific capacity of 327 mA h g^−1^ (capacity of first lithiation cycle) compared to 12C‐LIB (478 mA h g^−1^) and 14C‐LIB (388 mA h g^−1^). Despite its lower capacity, 16C‐LIB demonstrated a higher initial coulombic efficiency (ICE) of 60.5%, which is notably superior to that of 12C‐LIB (44.6%) and 14C‐LIB (46.4%). This suggests more significant electrolyte decomposition during the initial cycle in 12C‐LIB and 14C‐LIB (the shoulder‐like plateau observed in the discharge curve around 0.7 V can be attributed to electrolyte decomposition and interfacial layer formation during the first cycle). In the case of Na‐ion batteries, 14C‐NIB delivered the highest specific capacity of 400 mA h g^−1^ (capacity of first sodiation cycle), outperforming 12C‐NIB (298 mA h g^−1^) and 16C‐NIB (179 mA h g^−1^). However, despite its higher capacity, 14C‐NIB exhibited a lower ICE of 48%, compared to 58.5% for 12C‐NIB. Nonetheless, over‐extended cycling, 14C‐NIB outperformed 12C‐NIB, achieving a capacity retention of 42.7% after 100 cycles. However, in a head‐to‐head comparison, 16C‐LIB outperforms 14C‐NIB, demonstrating an ICE of 60.5% and a capacity retention of 60.2% after 100 cycles. The charge–discharge profiles of all anodes obtained from electrochemical conditioning for both types of ions were identical. This is because carbon consists of an ordered‐disordered microstructure at the nano scale, providing electronically and geometrically heterogeneous sites. The charging–discharging profiles were divided into two characteristic regimes in accordance with the “adsorption–intercalation/filling” model [30, 31]: a sloping region (>0.1 V) and a plateau region (0.1–0.01 V). In both Li and Na systems, the sloping segment is attributed to adsorption processes. In contrast, the plateau region exhibits system‐specific mechanisms: in the Li system, it reflects combined ion intercalation alongside progressive pore filling, whereas in the Na system, it predominantly signifies pore‐filling phenomena. Ion anchoring occurred across the entire voltage spectrum (from 3.0 to 0.01 V), resulting in a discharge profile with a long sloping region, without any obvious staging method. For LIBs, the offered capacity from the high‐potential region (>0.1 V vs. Li^+^/Li) and the low‐potential region (0.1–0.01 V) both increased with in‐plane micro‐ordering in the carbon. This was due to the micro‐ordering of phases and the micro‐fusion of the pore network, which was heterogeneously distributed within the carbon. However, for Na cells, the capacity initially increased (from 12C‐NIB to 14C‐NIB) and then decreased (in 16C‐NIB). This behavior is directly related to the ionic radius of Na^+^ (0.102 nm), which is approximately 34.2% larger than that of Li^+^ (0.076 nm). When structural micro‐ordering occurred in the carbon due to thermal molecular rearrangement, it resulted in more ordered domains but reduced the potential for rapid ion diffusion. Consequently, smaller ions like Li^+^ were able to anchor to more sites in the host, while larger ions like Na^+^ encountered barriers to diffusion. The differential capacity plots for lithiation and sodiation during the first, second, and 100th cycles are shown in Figures S7 and S8. The initial intercalation cycle reveals that all carbon electrodes exhibit two distinct peaks: one around 1.25 V and the other between 0.04–0.5 V. The peak at 1.25 V corresponds to electrolyte decomposition, while the broader peak at 0.04–0.5 V is attributed to the formation of the solid electrolyte interphase (SEI) on the electrode surface [8]. Notably, these reduction peaks disappear in the second cycle, signifying that the side reactions (electrolyte decomposition and SEI formation) are the primary contributors to the initial irreversible capacity loss of the cells. The cyclic performance of the carbon materials was evaluated at current densities ranging from low (50 mA g^−1^) (Figure 3g,h) to high (500 mA g^−1^) (Figure 4a,c) to further elucidate their electrochemical proficiency. The results indicate that the carbon exhibits superior performance in Li‐ion batteries compared to Na‐ion batteries. In addition to cyclic testing, the rate capability (Figure 4b,d) of the carbon materials was also assessed across a wider range of current densities (50 to 1000 mA g^−1^). Consistent with the cyclic performance results, the rate capability analysis further corroborates the superior electrochemical behavior of carbon in Li‐ion systems compared to Na‐ion systems. In Li‐ion batteries, among all tested samples, CD‐16C demonstrated the best rate performance, delivering charge capacities of 253.7–239.3, 217.2–216.6, 147.3–164.2, and 140.1–146.6 mA h g^−1^ at current densities of 0.05, 0.1, 0.5, and 1 A g^−1^, respectively. Notably, when the current density was returned to 0.05 A g^−1^, CD‐16C maintained an average charge capacity of 244.8‐240.7 mA h g^−1^, indicating excellent structural stability and rate capability. In contrast, for Na‐ion batteries, the sample CD‐14C exhibited the best rate performance, achieving charge capacities of 188.1–164.3, 128.5–110.2, 68.3–68.1, and 53.1–52.6 mA h g^−1^ at the same current densities. Upon reverting the current density to 0.05 A g^−1^, CD‐14C retained an average charge capacity of 155.4–139.2 mA h g^−1^, further underscoring its robust structural integrity and promising rate performance as an anode material for Na‐ion batteries. Furthermore, the cyclic stability of the reported carbon anode in the symmetric cell for an extended period has been illustrated in (Figures S9 and S10). A comparative analysis of the reversible capacities of our materials and the reported carbon anode is summarized in Table S6. Literature reports indicate that most carbon materials exhibit specific capacities in the range of 200–400 mA h g^−1^ at current densities below 40 mA g^−1^. In contrast, our highlighted samples (CD‐14C‐NIB and CD‐16C‐LIB) achieve reversible capacities of 190–200 mA h g^−1^ at a current density of 50 mA g^−1^ in coin cell testing a considerably higher current density, which emphasizes their strong performance. Furthermore, Sections 2.4 and 3 provide a detailed correlation between the electrochemical behaviors of these carbon electrodes and their unique hybrid microstructure. The electrochemical performance of electrode materials is strongly influenced by their electrical conductivity. As presented in Figures S11–S13, the conductivity of carbon samples (measured using an Ossila Four‐Point Probe), demonstrates a clear trend of increasing conductivity with rising temperature. This enhancement in conductivity is attributed to the thermal reduction of structural impediments, such as oxygen‐containing functional groups, hydrocarbon fragments, and sp^3^‐hybridized carbon atoms, which typically hinder electron transport [32]. Elevated temperatures facilitate the removal of these groups, thereby improving the material's conductive pathways. Specifically, the conductivity values for the carbon samples CD‐12C, CD‐14C, and CD‐16C are approximately 934 S·m^−1^, 1.3 kS·m^−1^, and 1.66 kS·m^−1^, respectively. This progressive increase is likely due to the collapse of micropores, formation of mesopores, increase in sp^2^ carbon content, and elimination of surface functional groups, all contributing to a more ordered carbon structure. Consequently, the superior electrochemical performance of CD‐16C in lithium‐based systems can be directly linked to its high conductivity, which stems from a greater degree of structural ordering and enhanced electron mobility.

To assess the electrochemical performance of the carbon materials employed as anode active components, long‐term cycling tests were carried out at a high current density. Consequently, it is essential to assess the structural properties of the electrode materials following extended cycling. Post‐cycling material characterization was performed using X‐ray diffraction (XRD; Figures S14 and S15) and Raman spectroscopy (Figures S16 and S17). The sharp diffraction peaks observed at 43.5°, 50.5°, and 74.1° in Figures S14a and S15a are attributed to the copper (Cu) foil, which serves as the current collector. A detailed examination of the zoomed‐in XRD patterns (Figures S14b–d and S15b–d) before and after cycling reveals the appearance of several new diffraction peaks. The peak around 20.5° corresponds to the polyvinylidene fluoride (PVDF) binder (ICDD 00‐061‐1404), while the minor peaks at 30.5°, 31.6°, 33.6°, and 38.7° are associated with carbonate (CO_3_^2^^−^), oxalate (C_2_O_4_^2^^−^), phosphate (PO_4_^3^^−^), and fluoride (F^−^) species, respectively. These compounds are likely formed due to electrolyte decomposition during the initial cycles and the subsequent development of the solid electrolyte interphase (SEI) layer on the carbon surface during electrochemical operation [14].  Regarding the structural integrity of the electrode material, the broad hump‐shaped peak around 24° remains nearly unchanged before and after cycling. It is important to emphasize that the carbon used in this study is hybrid in nature, comprising both ordered and disordered phases, as previously discussed in Sections 2.1 and 2.2 (refer to HR‐TEM images in Figure 1g–i and XRD in Figure 2C). Given the inherent amorphous characteristics of the carbon, minor structural changes induced by long‐term cycling are unlikely to significantly affect the broad peak around 24°. The XRD results of the post‐cycled carbon (Figures S14b–d and S15b–d) confirm that the broad peak at 24° remains identical with that of the pristine carbon, suggesting that the material has retained its structural integrity after 500 cycles at a high current density of 500 mA g^−1^. This conclusion is further corroborated by Raman spectroscopy. The Raman analysis (Figures S16 and S17) indicates that the carbon structure remains largely unchanged. The band intensity ratios for CD‐12C, CD‐14C, and CD‐16C were 1.02, 1.13, and 1.21, respectively, prior to electrochemical cycling. Post‐cycling characterization reveals that after 500 cycles at a current density of 500 mA h g^−1^, the band ratios are 1.01–1.04, 1.14, and 1.20–1.21 for CD‐12C, CD‐14C, and CD‐16C, respectively, which are almost identical to those of the raw materials. This consistency in Raman band ratios strongly suggests that the carbon structure retained its integrity even after prolonged cycling under elevated current densities.

The electrochemical impedance spectrum (EIS) of the materials after 100 cycles are presented in Figure 4e,f. Each Nyquist plot displays a semicircle in the high‐frequency region, corresponding to the charge transfer resistance (R_ct_) at the interface, and a straight line in the low‐frequency region, which represents the ion diffusion impedance (R_s_) within the bulk electrode [33]. Why is R_ct_ important in electrochemical kinetics? R_ct_ is a critical parameter because it directly reflects the rate of the redox reactions occurring at the electrode surface. A lower R_ct_ indicates more efficient electron transfer, which is essential for high‐performance electrochemical systems. In this study, the same material exhibits different R_ct_ values depending on the electrochemical system in which it is tested. Specifically, CD‐16C‐LIB shows the lowest R_ct_ in the lithium system, while CD‐14C‐NIB exhibits the lowest R_ct_ in the sodium system. This suggests superior solid electrolyte interphase (SEI) layer formation on CD‐16C in the Li system and on CD‐14C in the Na system [34, 35] which aligns with the excellent rate capability and cycling stability observed for these electrodes. Moreover, CD‐16C‐LIB demonstrates the lowest R_ct_ among all tested electrodes, indicating the most favorable electron transfer kinetics. This can be attributed to its excellent ion diffusion behavior, as further supported by GITT analysis.

### 2.4 Nano Pore Evolution From In‐Plane Micro‐Ordering and Ion Diffusion Kinetics

**FIGURE 5:**
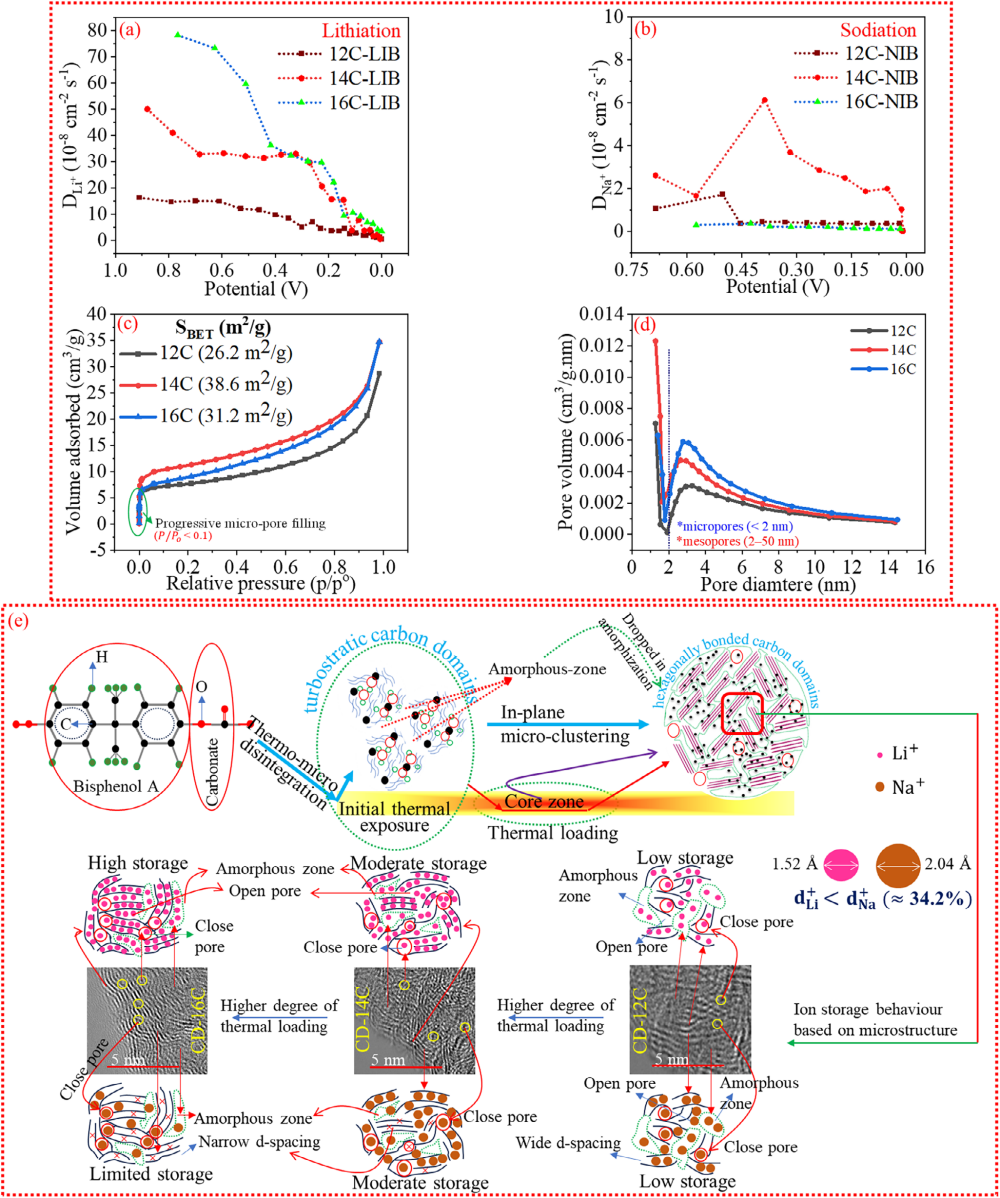
(a,b) Calculated diffusion coefficients of Li^+^ during lithiation and Na^+^ during sodiation in carbon materials, highlighting ion transport behavior. (c) Nitrogen adsorption–desorption isotherms of carbons carbonized at various temperatures, indicating surface area and porosity characteristics. (d) Pore size distributions corresponding to the samples in (c), illustrating the evolution of micro‐ and mesoporous structures. (e) Schematic representation of the microstructural evolution within the hybrid carbon matrix as carbonization temperature increases, emphasizing the role of localized nanostructural disorder in enhancing ion storage performance.

Through the Galvanostatic Intermittent Titration Technique (GITT), a comparative analysis of the diffusivity of Li^+^ (D_Li_^+^) and Na^+^ (D_Na_^+^) ions during electrochemical conditioning of carbon materials in Li and Na systems was performed. The GITT measurements were conducted from the second charge–discharge cycle, applying a pulse current of 50 mA g^−1^ for 15 min, followed by a 2 h rest period to allow the voltage to stabilize. The GITT profiles are presented in (Figures S18 and S19), with the corresponding diffusion coefficients shown in Figure 5a,b. In the Li system, D_Li_^+^ increases with in‐plane micro‐ordering of the carbon structure. In contrast, for the Na system, the diffusivity behavior is the opposite: D_Na_^+^ decreases with increasing in‐plane micro‐ordering. Nevertheless, for both chemistries, the diffusion coefficient gradually declines across all carbon samples. In both Li‐ion and Na‐ion batteries, at higher potentials, ion adsorption is favored at more energetically stable sites, such as the edges of microdomains and interlayer sites within the in‐plane ordered region. Once these anchoring sites become occupied, ion diffusion from these adsorption sites to regions with lower energy favorability is slowed, leading to ion congestion. This effect results in diminished insertion kinetics. This kinetic phenomenon aligns with the observed decrease in the diffusion coefficient (D_M_^+^) below 0.3 V, indicating that ion anchoring becomes less favorable at lower voltages. During de‐lithiation (Li) (Figure S18c,f,i) and de‐sodiation (Na) and (Figure S19c,f,i), the diffusivity gradually declines with increasing potential. Ions are initially released from low‐energy sites at low potential and move out of the carbon microstructure. At higher potentials, diffusion associated with the de‐anchoring process from energetically favorable sites proceeds at a slower pace, contributing to the observed decrease in the diffusion coefficient. The diffusion kinetics of Li^+^ and Na^+^ ions during intercalation and deintercalation processes are strongly influenced by the microstructure of the host material, and it is carbon in this study. Among various structural features, the porosity of the carbon plays a pivotal role in facilitating and regulating ion transport dynamics.

Therefore, the porosity of the carbons was evaluated using nitrogen (N_2_) adsorption and desorption isotherms. As illustrated in Figure 5c,d, the isotherms demonstrate an increasing adsorption trend with rising relative pressure (p/p°), indicating the nanoporous microstructure of the carbons. The BET‐specific surface area of the hard carbons was determined to be 26.2, 38.6, and 31.2 m^2^/g, respectively. Notably, the BET surface area initially increases and subsequently decreases with rising temperature. This trend can be attributed to the formation of micropores at lower temperatures, followed by their coalescence or fusion at higher temperatures. Furthermore, high‐temperature thermal treatment leads to the removal of oxygen‐containing functional groups, facilitating structural rearrangement of the carbon [22]. This microstructural ordering may also contribute to a reduction in the surface area. The observation of micropore evolution and fusion is further assessed by the pore size distribution analysis of the carbons, as presented in Figure 5d. For the sample CD‐12C, micropores (<2 nm) constitute the dominant pore phase. In contrast, the pore size distribution of CD‐14C reveals a decrease in micropore volume accompanied by an increase in mesopore (2–50 nm) volume. Meanwhile, CD‐16C exhibits a smaller pore size distribution, with mesopores emerging as the dominant phase. The reduction in pores at high‐temperature thermal treatment can be attributed to the fusion of micropores into mesopores, accompanied by atomic rearrangements that promote the growth and expansion of hexagonally bonded sp^2^ carbon domains through micro‐ordering and structural reorganization. Interestingly, such porous structures are advantageous for ion storage applications, as they facilitate both pore filling and efficient ion diffusion through the porous network, enhancing their potential for energy storage [22]. The diffusion kinetics analysis (Figure 5a,b) indicates that the hybrid‐structured carbon demonstrates superior electrochemical performance in the Li system compared to sodium‐based ones. This enhanced behavior can be primarily attributed to the fundamental differences in the physical properties of Li^+^ and Na^+^ ions. Specifically, the ionic radius of Na^+^ is approximately 34% larger than that of Li^+^, which leads to significantly slower interparticle diffusion of Na^+^. In contrast, the smaller Li^+^ ions can diffuse more rapidly and readily access ultra‐micropores (d <0.7 nm), where they can be efficiently localized, thereby improving ion storage capability. It is important to note that, in addition to open pores (characterized by N_2_ adsorption/desorption isotherms), closed pores (identified through HR‐TEM images) also play a significant role in electrochemical kinetics via closed‐pore filling. While the volume of open pores tends to decrease due to pore fusion caused by inter‐pore wall coalescence, both the volume and size of closed pores increase with high‐temperature thermal treatment. The assessment of closed pores in the carbon materials using HR‐TEM is summarized in Figure 5e. While the precise quantification of closed pores remains challenging, HR‐TEM imaging offers valuable topographical insights, allowing visual identification of such features. At lower temperature, closed pores are scarcely observable, as the carbon matrix appears dense and comprises randomly oriented, fragmented graphene sheets. With increasing thermal treatment, near‐spherical voids encased by short stacks of graphene layers become more prominent. These features are particularly distinct in CD‐16C, where the internal microstructure demonstrates higher ordering. This enhanced structural arrangement facilitates efficient ion diffusion in Li systems. However, CD‐16C does not exhibit comparable performance in Na batteries. This discrepancy arises because ion transport kinetics are influenced not only by pore architecture—whether open or closed—but also by the interlayer (d‐) spacing within the microdomains. It is anticipated that the narrower d‐spacing in CD‐16C though facilitated Li^+^ diffusion but was less favorable for the larger Na^+^ ions. Figure 5e schematically illustrates the microstructural evolution of carbon as a function of increasing thermal transformation temperature, along with the corresponding ion storage behavior of the hybrid‐carbon matrix in both lithium and sodium systems. Moreover, the electrochemical performance of carbon materials is closely correlated with their microstructure through theoretical studies, as the hybrid nature of the reported carbon significantly influences the storage behavior of the ions. For Na^+^ ions, a wide interlayer spacing is preferred due to their large ionic radius; theoretical studies indicate that when the graphite layer spacing is less than 0.353 nm, Na‐ion insertion requires considerable energy, making it challenging, whereas at 0.36 nm the energy dissipation drops to −0.159 eV, enabling insertion, and at ∼0.40 nm the energy consumption falls below −0.698 eV [36, 37]. Among the studied carbons, CD‐16C exhibits the smallest average d‐spacing (0.36 nm), while CD‐12C and CD‐14C show larger spacings of 0.402 and 0.373 nm, respectively, allowing easier Na‐ion insertion into their graphitic domains and consequently higher plateau capacities for CD‐12C‐NIB and CD‐14C‐NIB compared to CD‐16C‐NIB. Furthermore, defective and ultra‐microporous carbons exhibit Na^+^ adsorption energies of −1.996 and −1.598 eV, respectively, which are lower than that of defect‐free carbon (−1.195 eV) [38], suggesting that the defective and porous nature of the reported carbons likely results in adsorption energies below −1.195 eV, correlating with their higher ion storage capacity and specific capacity. For Li storage, the binding energy (E_B_) between an absorbed Li atom and graphene sheets is calculated as −0.95 eV, indicating a stronger interaction than Na^+^, with the most stable sites located at nanopore defect centers [39]. CD‐12C, CD‐14C, and CD‐16C possess high surface areas (26.2, 38.6, and 31.2 m^2^/g) and open/closed pores, enhancing electron‐accepting tendencies of their defective nanoarchitecture, which facilitates electron gain from Li atoms. Compared to pristine graphene sheets, these defective structures exhibit significantly improved binding energy (−3.17 eV), demonstrating that the Li adsorption capability of nano graphitic domains is greatly enhanced by porosity, and the more negative E_B_ of defective graphene sheets provides direct evidence of the synergistic effect of nanopore defects for Li storage [39].

## 3 Local Microstructural Features and Material Performance

Based on the structural morphology analysis, it is evident that the reported carbons are neither fully ordered nor entirely disordered but rather exhibit a hybrid structure combining features of both. Furthermore, electrochemical characterization reveals that these carbons exhibit distinct behaviors in Li and Na systems. This clearly demonstrates that the structural morphology of carbon plays a critical role in governing ion storage mechanisms during charge and discharge processes. In this section, we directly correlate the electrochemical performance of the carbons with their localized microstructural characteristics.

**FIGURE 6:**
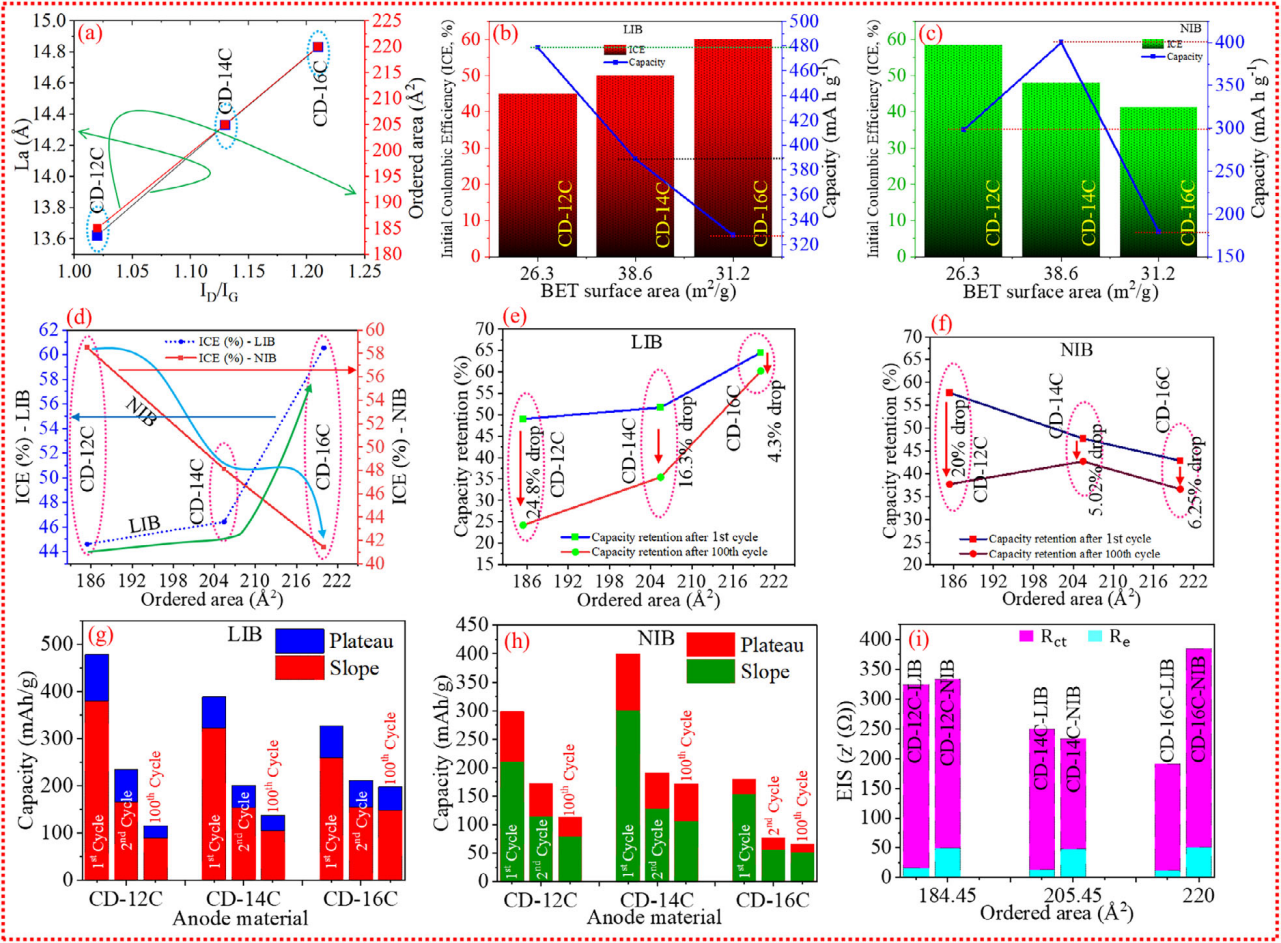
Comprehensive analysis of local microstructural disorder and its impact on electrochemical performance. (a) Relationship between Raman band ratio, crystallite size, and ordered domain size. (b) Correlation of BET surface area with initial Coulombic efficiency (ICE) and initial lithiation capacity in lithium‐ion systems. (c) Correlation of BET surface area with ICE and initial sodiation capacity in sodium‐ion systems. (d) Dependence of ICE on ordered domain size. (e) Influence of ordered domain size on capacity retention in lithium‐ion systems. (f) Influence of ordered domain size on capacity retention in sodium‐ion systems. (g) Evolution of sloping and plateau capacities over the first, second, and 100th lithiation cycles for lithium‐ion battery (LIB) anodes. (h) Evolution of sloping and plateau capacities over the first, second, and 100th sodiation cycles for sodium‐ion battery (NIB) anodes. (i) Correlation between ordered domain size and charge transfer resistance (Rct) and electrolyte resistance (Re).

Raman analysis, which reveals the local structural order and defects in carbon materials, indicates that the band ratio of the carbon increases with high‐temperature carbonization. Using this band ratio, the ordered area within the carbon structure was calculated using Equation (2). Figure 6a illustrates the correlation between La and the ordered domain as a function of the Raman band ratio. It shows that CD‐16C exhibits a larger La and ordered domain area (in Å^2^), while CD‐12C displays a smaller La and domain area. The specific capacity analysis of Li‐ion half‐cells reveals that although CD‐12C has a smaller La and domain area, CD‐16C—with its larger La and domain area—exhibits better cycling stability among all tested half‐cells. Furthermore, X‐ray PDF analysis (Figure 2g,h) supports the Raman findings. CD‐16C shows a slower decay in C–C correlations, consistent with a larger aromatic carbon domain and less localized structural disorder. These X‐ray PDF results provide additional evidence that local structural disorder is directly correlated with electrochemical performance. Porosity in carbon has long been considered a key factor influencing surface area, which plays a critical role in ion storage capacity and ICE. Figure 6b shows that although CD‐12C has the lowest surface area, it delivers the highest initial capacity but suffers from low ICE in the Li system. Conversely, CD‐16C, with a high surface area, shows a lower initial capacity but achieves the highest ICE. Interestingly, in sodium‐ion systems (Figure 6c), the more defective carbon CD‐12C provides the highest ICE, while CD‐14C—with an intermediate ordered domain size—delivers the highest initial capacity. These findings suggest that surface area and porosity alone are not sufficient to govern ICE and capacity. In this context, local/micro‐regional structural defects and the size of hexagonally bonded aromatic carbon domains must be considered when interpreting ICE and capacity behavior. Figure 6d shows that in Li systems, ICE increases with increasing domain size. However, in Na systems, the opposite trend is observed: ICE decreases as the domain size increases. Despite this, CD‐16C in the Li system provides the highest ICE among all samples. Although CD‐12C—the most defective carbon—exhibits the highest ICE in the Na system, it fails to maintain stable cycling performance. As shown in Figure 6f, CD‐12C retains the highest capacity initially in the Na system, but its capacity drops by 20% after 100 cycles. In contrast, CD‐14C experiences only a 5.02% capacity loss after 100 cycles. This indicates that while a high volume of defects can provide high initial capacity, carbon with a moderate level of order (≈ 205 Å^2^) and fewer defects ensures better long‐term capacity retention. These findings are even more apparent in the Li system (Figure 6e). CD‐12C, the most defective carbon, shows a 24.8% drop in capacity retention, whereas CD‐16C—with a larger ordered domain and fewer defects—exhibits the lowest drop at 4.3%, making it the most stable among all samples across both systems. This highlights the importance of understanding the hybrid nature of carbon and its electrochemical kinetics, especially when comparing different electrochemical systems. It is evident that structural defects play a critical role in boosting initial capacity, particularly at high voltage regions (>0.1 V, the sloping region). Table S7 summarizes the carbon microstructure and electrochemical performance, highlighting dominant ion‐storage mechanisms in Li‐ion and Na‐ion systems as a function of structural characteristics. Figure 6g,h shows that most of the capacity originates from the sloping region, while the plateau capacity contribution is limited. However, with extended cycling, this dynamic changes: sloping capacity drops significantly, while plateau capacity remains relatively stable. This suggests that the high‐voltage region (>0.1 V) is primarily responsible for SEI layer formation and electrolyte decomposition via irreversible chemical reactions. Histograms of sloping and plateau capacities reveal that in the Li system, CD‐16C delivers the highest sloping and plateau capacities at the 100^th^ cycle. This implies that CD‐16C offers more reversible ion storage sites across both high (>0.1 V) and low (<0.1 V) voltage regions compared to CD‐12C and CD‐14C. Conversely, in the Na system, CD‐14C provides the highest sloping and plateau capacities at the 100th cycle, suggesting it has more reversible storage sites than CD‐12C and CD‐16C in that system. Thus, it is evident that the same carbon material can behave differently depending on the electrochemical environment. Two key factors can be hypothesized to explain this behavior: (i) the local/micro‐regional structural features of the material, and (ii) the physical and/or chemical characteristics of the ion systems. This influence becomes even more pronounced when analysing the EIS data (Figure 6i), which shows that the electrolyte resistance (R_e_) is higher in Na systems than in Li systems. Future research could focus on developing more stable and compatible electrolytes for sodium‐ion batteries to reduce R_e_ and improve performance.

Overall, although the carbon materials exhibit comparable ion‐storage capacities, their underlying ion storage mechanisms differ significantly. This variation highlights a predominant dependence on the hybrid carbon matrix structure of the carbon, particularly influenced by the presence of structural defects. Electrochemical kinetics analysis identifies CD‐16C‐LIB as the most efficient among the tested carbons. In direct comparisons, the synthesized carbon—characterized by a localized hybrid microstructure combining ordered and disordered carbon—demonstrates superior performance in lithium‐ion (Li^+^) systems relative to sodium‐ion (Na^+^) systems. This disparity is attributed to the intrinsic physical properties of the Na^+^ ion, which critically influence electrochemical kinetics. Specifically, increased structural ordering appears to hinder Na^+^ diffusion (D_Na^+^), whereas Li^+^ diffusion (D_Li^+^) consistently outpaces D_Na^+^ under all tested conditions. This results in enhanced electrochemical performance for Li^+^ systems. Furthermore, according to the BET analysis (Figure 5c,d), the carbons exhibit abundant micro‐ and mesopores, where mesopores serve as pathways for electrolyte ions to access internal micropores, enhancing ion transport. The TEM analysis (Figure 5c) further reveals the presence of closed pores. These hierarchical pores provide a highly accessible reaction area, which is crucial for achieving high energy storage in LIBs and SIBs, while also buffering large volume changes during electrochemical reactions, resulting in high gravimetric capacity and good rate performance [40]. However, it is not only the open pore that contribute to the plateau capacity, it is also the closed pores which contribute to the plateau capacity [41]. Importantly, both open and closed pores contribute to the low‐voltage (0.1–0.01 V) plateau capacity via a pore‐filling mechanism. The initial lithiation plateau capacities for CD‐12C, CD‐14C, and CD‐16C are 98.5, 65.5, and 68.278 mA h g^−1^, respectively, but after 100 cycles, these values decrease to 27.3, 32.8, and 49.84 mA h g^−1^, with CD‐16C‐LIB retaining the highest plateau capacity due to its larger mesopore volume, higher closed‐pore content, and more graphitic domains compared to the others. The higher initial plateau capacity of CD‐12C‐LIB is attributed to its abundant micropores, as shown in Figure 5d, which also explains its lowest ICE (44.6%)—small micropores trap ions, boosting initial capacity but reducing ICE. In the Na system, CD‐14C‐NIB delivers the highest plateau capacity (64.74 mA h g^−1^) after 100 cycles, outperforming CD‐12C‐NIB (32.7 mA h g^−1^) and CD‐16C‐NIB (13.23 mA h g^−1^). Despite CD‐16C having the most mesopores and closed pores, its Na performance is poor because graphitic domains also influence low‐voltage capacity, and CD‐16C's smaller interlayer spacing (0.36 nm) is less favorable for Na^+^ storage compared to CD‐12C (0.402 nm) and CD‐14C (0.373 nm). Electrochemical kinetics assessed via GITT (Figure 5a,b) show Li^+^ ions diffuse faster than Na^+^ ions due to their smaller radius (∼0.76 Å vs. Na^+^ ∼1.02 Å). CD‐16C‐LIB exhibits the highest Li^+^ diffusion coefficient (*D*_*Li* +_) in the 0.1–0.01 V range, from 10.5 × 10^−^^8^ to 4.0 × 10^−^^8^ cm^−2^ s^−1^, which is 2.77–2.07 times greater than CD‐14C‐LIB and 4.37–2.1 times greater than CD‐12C‐LIB. In the Na system, CD‐14C‐NIB shows the highest diffusion coefficient at 0.1 V (1.87 × 10^−^^8^ cm^−2^ s^−1^), 5.2 times higher than CD‐12C‐NIB and 13.34 times higher than CD‐16C‐NIB, while all Na anodes stabilize at ∼0.11 × 10^−^^8^ cm^−2^ s^−1^ at 0.01 V. Overall, CD‐16C‐LIB demonstrates superior Li performance due to its mesopores, closed pores, and graphitic domains, whereas CD‐14C‐NIB excels in Na systems owing to its balanced mesopores, closed pores, and moderate graphitic domains. In the plateau region, capacity is jointly governed by open pores, closed pores, and graphitic domains; neglecting any of these features can lead to misinterpretation of electrochemical kinetics. Therefore, it is essential to consider all these microstructural features collectively when correlating electrochemical performance with diffusion kinetics.

## 4 Technoeconomic Evaluation and Environmental Impact Assessment of the Carbonization Process

One of the primary objectives of this study was to synthesize carbon material from waste, specifically utilizing discarded CDs as the feedstock for carbon preparation. In the context of upcycling waste for advanced applications, it becomes critically important to evaluate the technoeconomic aspect and environmental impact of the process.

Waste CDs were processed into carbon using a single‐step selective thermal transformation technique, a straightforward approach from a techno‐economic perspective (refer to the Supporting Information for detailed). Since carbonization is a thermal process, a 0.5‐ton Rotary Carbonization Furnace rating 22 kW was modeled to simulate large‐scale production, while a Horizontal Tube Furnace rating 6 kW was used for lab‐scale synthesis. Cost calculations (Tables S8–S11) considered key consumables such as electricity and nitrogen, with raw material cost assumed to be zero as waste CDs have no market value. Baseline prices included electricity at 0.267–0.45 AUD per kWh and nitrogen at approximately 1 AUD per kilogram. Based on this analysis, the production cost of carbon from waste CDs is estimated at 76–165 AUD per gram at the lab scale and approximately 6–10 AUD per kilogram at a large scale. Techno‐economic evaluations indicate that laboratory‐scale production incurs significantly high costs, primarily because only small quantities of material often just a few grams are processed. In contrast, scaling up the carbonization process to a 0.5‐ton capacity furnace results in a substantial reduction in cost. Future research will focus on comprehensive process optimization to further minimize production expenses. To this end, a comprehensive Life Cycle Assessment (LCA) was conducted to quantify the environmental footprint associated with the carbonization process (refer to the Supporting Information for detailed LCA results). The assessment was performed using SimaPro 9.6.0.1, a widely recognized and robust LCA software tool. The analysis indicates that, at the laboratory scale, electricity consumption is the dominant contributor to environmental impact. However, in scale‐up studies, the carbonization process itself emerges as the primary contributor. A direct comparison between lab‐scale and scaled‐up processes reveals a significant reduction in environmental impacts upon scaling (The impact categories and their derived values have been summarized in Table S13). Specifically, the lab‐scale carbonization process contributes approximately 163 815.3–211 993.4 kg CO_2_‐equivalent per kilogram of carbon produced from waste CDs, whereas the scaled‐up process contributes only 3.94–5.5 kg CO_2_‐equivalent per kilogram of carbon, representing a dramatic decrease in greenhouse gas emissions. In terms of damage assessment, the lab‐scale process impacts on resources, human health, and ecosystems were approximately 0.2–0.3, 7.4–9.6, and 1.2–1.5 kPt, respectively (as shown in Figures S21b, S22b, and S23b). In contrast, these values drop significantly in the scaled‐up process to 2.8–3.5, 92.5–115.7, and 15.0–18.8 mPt, respectively (refer to Figures S21d, S22d, and S23d). The LCA results clearly demonstrate that scaling up the process substantially reduces its environmental impact. Nevertheless, further research is warranted to optimize key process parameters such as temperature and duration, which could lead to additional reductions in environmental burden and enhance the overall sustainability of the carbonization process.

## 5 Conclusion

In this study, we aimed to elucidate how the hybrid microstructure of carbon electrodes influences their capacitive energy storage performance in lithium‐ion (Li‐ion) and sodium‐ion (Na‐ion) systems. Electrochemical measurements conducted on carbon materials synthesized across a broad temperature range (1200°C–1600°C) revealed a clear correlation between storage capacity and the physicochemical properties of the carbon. Our findings indicate that micro‐regional structural features—previously considered advantageous primarily for Na‐ion storage—exhibit superior performance in Li‐ion systems. This enhanced performance is likely attributed to a combination of factors, including the formation of larger hexagonally bonded carbon domains via phase clustering, the annealing of structural defects, and the elimination of surface functional groups during high‐temperature annealing. Notably, carbons with progressively ordered domains demonstrated improved long‐term storage capacity in Li‐ion batteries compared to Na‐ion batteries. We attribute this to the more efficient ion accommodation within the hybrid carbon matrix, which is better suited to the smaller ionic radius and higher mobility of lithium ions. Overall, this work highlights a previously underappreciated microstructural parameter—namely, the hybrid configuration of carbon—as a key determinant of superior electrochemical performance in Li‐ion systems. These insights offer valuable guidance for the rational design and synthesis of high‐performance, sustainable electrode materials tailored for rechargeable Li‐ion and Na‐ion batteries.

## 6 Experimental Section

### 6.1 Material Synthesis

In this research study, crushed compact discs (CDs) were utilized as a carbon‐based precursor material. The carbonization process was conducted in a horizontally aligned furnace equipped with single‐bore high alumina ceramic tubes, which served as thermal energy accumulators. These ceramic tubes facilitated the selective carbonization of the crushed CDs by storing and redistributing heat efficiently. The thermal transformation of the raw material proceeded through three distinct material transition phases within a single‐step carbonization process: Initially, the crushed CD material underwent thermal preconditioning at the lower‐temperature zone near the mouth of the high alumina ceramic tubes. This stage prepared the material for subsequent thermal reactions by initiating structural relaxation and partial volatilization. In the intermediate zone, the preconditioned material was exposed to temperatures ranging from 500°C to 600°C. This induced thermal depolymerization, breaking down the polymeric matrix of the CDs into a heterogeneous carbonaceous residue. Finally, the carbon residues were subjected to high‐temperature thermal annealing between 1200°C and 1600°C. This step facilitated the transformation of the heterogeneous carbon into a hybrid carbon material through selective carbonization and structural reorganization.

Each batch of the carbonization process utilized 2 g of crushed CDs as feedstock, resulting in the production of 0.36 to 0.4 g of carbon material. This corresponds to a carbonization yield ranging from 18% to 20%. For the purposes of the Life Cycle Analysis (LCA), the evaluation was conducted using the pessimistic yield value of 18%.

### 6.2 Material Characterization

To comprehensively investigate the compositional, morphological, and structural properties of the carbon material, a suite of advanced characterization techniques was employed:

#### 6.2.1 Elemental Analysis (CHNSO)

The elemental composition, including carbon, hydrogen, nitrogen, sulfur, and oxygen content, was determined using an Elementar *varioMACRO cube* and *rapidOXY cube* analysers. This combustion‐based analysis provided quantitative insights into the elemental makeup of the carbon samples.

#### 6.2.2 X‐Ray Photoelectron Spectroscopy (XPS)

Surface chemical states and elemental composition were analysed using XPS at a take‐off angle of 90° under an ultrahigh vacuum (∼2 × 10^−^^9^ mbar). The system was equipped with a monochromated Al Kα X‐ray source (hν = 1486.68 eV). The C 1s peak at 284.5 eV was used as the reference for binding energy calibration.

#### 6.2.3 Raman Spectroscopy

Raman spectra were acquired using an *inVia Qontor Raman microscope* with 50× magnification. A 514 nm green diode laser and a 2400 lines/mm UV grating were employed. Spectral data were collected over the range of 100–3200 cm^−1^ with an exposure time of 10 s, using 5% laser power and five accumulations to enhance signal quality.

#### 6.2.4 Brunauer–Emmett–Teller (BET) Surface Area Analysis

Specific surface area and porosity were measured using a *Micromeritics TriStar 3020 II* system under a nitrogen atmosphere. Prior to analysis, approximately 0.1 g of each sample was degassed under vacuum for 30 min, followed by nitrogen purging for an additional 30 min to eliminate residual moisture.

#### 6.2.5 X‐Ray Diffraction (XRD)

Crystallographic structure was examined using a *PANalytical X'Pert MPD* diffractometer with Cu Kα radiation (λ = 1.5406 Å), operated at 40 kV and 40 mA. This analysis provided insights into the phase composition and degree of crystallinity.

Field emission scanning electron microscopy (FE‐SEM): An FEI Nova NanoSEM 450 with a Schottky field emission source was used for SEM analysis.

#### 6.2.6 Atomic Force Microscopy (AFM)

AFM measurements were conducted using a *Bruker Dimension ICON* scanning probe microscope (SPM) equipped with a *Nanoscope V* controller (software version 9.70). Surface topography of both corroded and uncorroded samples was examined in PeakForce Tapping mode using a SCANASYST probe. The scan area was set to 1 µm × 1 µm, with a scan rate of approximately 0.5–0.6 Hz and a peak force of ∼800 pN. Image resolution was maintained at 512 pixels per line. Feedback gain parameters were adjusted as needed to ensure optimal surface tracking during scanning. Acquired AFM images were processed and analyzed using *Gwyddion* software to extract quantitative surface features and visualize morphological differences.

#### 6.2.7 High‐Resolution Transmission Electron Microscopy (HR‐TEM)

Morphological and lattice structural features were visualized using a *JEOL JEM‐F200* HR‐TEM operated at 200 kV. Imaging conditions included a spot size of 1, tilt angle of 5°, and an emission current of 14 µA to capture high‐resolution microstructural details.

### 6.3 Electrochemical Measurement for LIB

To prepare the anode, carbon was blended with carbon black and polyvinylidene fluoride (PVDF) (both from AVT Services) in a weight ratio of 80:10:10. N‐methyl‐2‐pyrrolidone (NMP) was added dropwise to the mixture, which was then magnetically stirred for 48 h to form a homogeneous slurry. The slurry was uniformly layered onto copper foil to a thickness of approximately 150 µm. The coated foil was first dried overnight in a fume hood, followed by vacuum drying at 120°C for 12 h. To enhance adhesion between the active material and the current collector, the dried electrode sheet was pressed at 100 kN for 1 h using a flat‐plate press (MTI Corporation). Circular electrode discs (12 mm in diameter) were punched from the pressed sheet using a handheld punching tool. These discs were further dried in a vacuum oven at 120°C for 1 h before being transferred into an argon‐filled glove box for cell assembly. CR2032‐type half coin cells were assembled using the prepared electrodes as the working electrode and lithium metal chips as the counter/reference electrode. A 16 mm diameter glass fibre separator (Whatman, Grade GF/F) was used, and the electrolyte consisted of 1 M LiPF_6_ dissolved in a 1:1 volume ratio of ethylene carbonate (EC) and dimethyl carbonate (DMC). Electrochemical performance was evaluated using a Neware BTS3000 battery tester within a voltage window of 0.01–3.0 V. The applied current values were determined based on the mass loading of the active material on the electrodes. The average mass loading, ranging from 1.8 to 2.0 mg on a 12 mm diameter disk, corresponds to approximately 1.59–1.77 mg/cm^2^. Electrochemical impedance spectroscopy (EIS) was conducted using a Bio‐Logic VSP‐300 potentiostat over a frequency range of 100 kHz to 50 mHz, with an AC amplitude of 10 mV.

### 6.4 Electrochemical Measurement for NIB

To prepare the anode, carbon was blended with carbon black and polyvinylidene fluoride (PVDF) (both from AVT Services) in a weight ratio of 80:10:10. N‐methyl‐2‐pyrrolidone (NMP) was added dropwise to the mixture, which was then magnetically stirred for 48 h to form a homogeneous slurry. The slurry was uniformly layered onto copper foil to a thickness of approximately 150 µm. The coated foil was first dried overnight in a fume hood, followed by vacuum drying at 120°C for 12 h. To enhance adhesion between the active material and the current collector, the dried electrode sheet was pressed at 100 kN for 1 h using a flat‐plate press (MTI Corporation). Circular electrode discs (12 mm in diameter) were punched from the pressed sheet using a handheld punching tool. These discs were further dried in a vacuum oven at 120°C for 1 h before being transferred into an argon‐filled glove box for cell assembly. CR2032‐type half coin cells were assembled using the prepared electrodes as the working electrode and sodium metal chips as the counter/reference electrode. A 16 mm diameter glass fibre separator (Whatman, Grade GF/F) was used, and the electrolyte consisted of 1 M NaPF_6_ dissolved in a 1:1 volume ratio of ethylene carbonate (EC) and dimethyl carbonate (DMC). 2 vol% fluoroethylene carbonate (FEC) additive was incorporated into the electrolyte solution, the inclusion of FEC was to promote the formation of a stable solid electrolyte interphase (SEI) at the electrode–electrolyte interface, thereby enhancing the performance and stability of sodium‐ion batteries (NIBs). Electrochemical performance was evaluated using a Neware BTS3000 battery tester within a voltage window of 0.01–3.0 V. The applied current values were determined based on the mass loading of the active material on the electrodes. The average mass loading, ranging from 1.8 to 2.0 mg on a 12 mm diameter disk, corresponds to approximately 1.59–1.77 mg/cm^2^. Electrochemical impedance spectroscopy (EIS) was conducted using a Bio‐Logic VSP‐300 potentiostat over a frequency range of 100 kHz to 50 mHz, with an AC amplitude of 10 mV.

## Conflicts of Interest

The authors declare no conflicts of interest.

## Supporting information




**Supporting File**: advs74060‐sup‐0001‐SuppMat.docx.

## Data Availability

The data that support the findings of this study are available from the corresponding author upon reasonable request.
