# Supplementary material for: Tuning Li+ and Na+ Functionality in Renewable Carbon Electroactive Material Through Site‐Specific Nanostructural Disorder
Source: Adv Sci (Weinh). 2026 Jan 31;13(19):e23383. doi: 10.1002/advs.202523383 (PMC13045420; doi:10.1002/advs.202523383)
Supplement: Supplementary file 1 — Supporting File: advs74060‐sup‐0001‐SuppMat.docx. [file ADVS-13-e23383-s001.docx]

***Supporting Information***

**Tuning Li^+^ and Na^+^ Functionality in Renewable Carbon Electroactive Material Through Site-specific Nanostructural Disorder**

Montajar Sarkar**^*a^**, Rumana Hossain**^*a^**, Jian Peng**^b^**, Dilan Kumara Thilakarathna**^b^**, Yu Wang**^c^**, Yin Yao**^d^**, Neeraj Sharma**^b^**, Veena Sahajwalla**^a^**

^a^ Centre for Sustainable Materials Research and Technology, SMaRT@UNSW, School of Materials Science and Engineering UNSW Sydney, NSW 2052, Australia

^b^ School of Chemistry UNSW Sydney, NSW 2052, Australia

^c^ Solid State & Elemental Analysis Unit (SSEAU), X-ray Diffraction Laboratory, Mark Wainwright Analytical Centre, UNSW Sydney, NSW 2052, Australia

^d^ Electron Microscope Unit (EMU), Mark Wainwright Analytical Centre, UNSW Sydney, NSW 2052, Australia

*E-mail: [montajar.sarkar@unsw.edu.au](mailto:montajar.sarkar@unsw.edu.au); [r.hossain@unsw.edu.au](mailto:r.hossain@unsw.edu.au)

**Table S1.** Breakdown of Compositional and Structural Properties of CD-12C.

| Name | Peak BE | FWHM (eV) | Area (P) (CPS.eV) | Atomic % |
| --- | --- | --- | --- | --- |
| C1s A | 284.5 | 0.85 | 22338.69 | 85.06 |
| C1s B | 285.3 | 0.85 | 2786.13 | 10.61 |
| C1s C | 286.3 | 0.85 | 805.27 | 3.07 |
| O1s A | 531.4 | 2.51 | 925.14 | 1.26 |

**Table S2.** Breakdown of Compositional and Structural Properties of CD-14C.

| Name | Peak BE | FWHM (eV) | Area (P) (CPS.eV) | Atomic % |
| --- | --- | --- | --- | --- |
| C1s A | 284.5 | 0.81 | 26018.05 | 86.51 |
| C1s B | 285.3 | 0.8 | 2725.04 | 9.06 |
| C1s C | 286.22 | 0.8 | 981.27 | 3.26 |
| O1s A | 531.96 | 2.71 | 978.84 | 1.16 |

**Table S3.** Breakdown of Compositional and Structural Properties of CD-16C.

| Name | Peak BE | FWHM (eV) | Area (P) (CPS.eV) | Atomic % |
| --- | --- | --- | --- | --- |
| C1s A | 284.5 | 0.77 | 24659.61 | 86.78 |
| C1s B | 285.3 | 0.77 | 2855.25 | 8.13 |
| C1s C | 286.1 | 0.77 | 934.07 | 3.25 |
| O1s A | 532 | 2.3 | 831.96 | 1.03 |

**Table S4.** CHNSO analysis of waste CD and carbon (CD-12C, CD-14C, and CD-16C) synthesized from waste CD.

| **Sample** | **N (%)** | **C (%)** | **H (%)** | **S (%)** | **O (%)** |
| --- | --- | --- | --- | --- | --- |
| CD | 0.06 | 76.19 | 5.29 | ND | 19.4 |
| CD-12C | 0.65 | 92.87 | 0.71 | ND | 4.14 |
| CD-14C | 0.44 | 94.46 | 0.24 | ND | 2.01 |
| CD-16C | 0.10 | 97.34 | 0.09 | ND | 0.28 |

**ND = Not detected*

**Table S5.** Trace element content in waste CD and carbon (CD-12C, CD-14C, and CD-16C) synthesized from waste CD.

| **CD** | | | **CD-12C** | | | **CD-14C** | | | **CD-16C** | | |
| --- | --- | --- | --- | --- | --- | --- | --- | --- | --- | --- | --- |
| **Element** | **Conc.** | **Unit** | **Element** | **Conc.** | **Unit** | **Element** | **Conc.** | **Unit** | **Element** | **Conc.** | **Unit** |
| Al | 134 | ppm | Al | 0.12 | % | Al | 859 | ppm | Al | 623 | ppm |
| Si | 0.16 | % | Si | 1.08 | % | Si | 1.09 | % | Si | 1.19 | % |
| P | 0.18 | % | P | 0.15 | % | P | 0.15 | % | P | 0.17 | % |
| Cl | 0.10 | % | S | - | - | S | - | - | Cl | 430 | ppm |
| Ca | 0.11 | % | Cl | 309 | ppm | Cl | 314 | ppm | K | 501 | ppm |
| Ti | 0.52 | % | K | 0.13 | % | K | 0.13 | % | Ca | 0.80 | % |
| Cr | 160 | ppm | Ca | 0.86 | % | Ca | 0.92 | % | Ti | 6.46 | % |
| Zr | 0.66 | % | Ti | 5.68 | % | Ti | 6.22 | % | Cr | 530 | ppm |
|  |  |  | Cr | 0.10 | % | Cr | 0.10 | % | Zr | 0.23 | % |
|  |  |  | Zr | 0.26 | % | Zr | 0.25 | % | Ba | 578 | ppm |
|  |  |  | Ba | 694 | ppm | Ba | 611 | ppm | S | - | - |

**Table S6.** Electrochemical performance comparison of diverse carbon-based anode in alkali metal-ion batteries, including lithium-ion (LIB) and sodium-ion (SIB) systems.

| **Sample** | **Current density**  **(mA g^-1^)** | **LIB**  **Capacity (mA h g^-1^)** | **NIB**  **Capacity (mA h g^-1^)** |
| --- | --- | --- | --- |
| HC-1200C-2h [1] | 20 | - | 260 |
| HC-1400C-2h [1] |  | - | 300 |
| HC-1600C-2h [1] |  | - | 270 |
| G1000 [2] | 30 | $\sim$350 | $\sim$200 |
| G1300 [2] |  | $\sim$300 | $\sim$275 |
| G1500 [2] |  | $\sim$200 | $\sim$340 |
| G1700 [2] |  | $\sim$170 | $\sim$250 |
| G1900 [2] |  | $\sim$155 | $\sim$200 |
| o-HCS [3] | 37.2 | 420.5 | - |
| c-HCS [3] | 37.2 | 257.9 | - |
| c-HC (commercial HC; 1200C) [4] | 4 | 250.3 | 305.2 |
| f-HC (1800C) [4] |  | 317.6 | 276.7 |
| p-HC (1100C) [4] |  | 360.4 | 297.1 |
| **CD-12C (this work)** | **50** | **213.7** | **174.5** |
| **CD-14C (this work)** |  | **180.4** | **192.2*** |
| **CD-16C (this work)** |  | **200*** | **74.3** |
| * Only 4.3% drop in capacity retention after 100 cycles  * 5.02 % drop in capacity retention after 100 cycles | | | |

**Table S7:** Summary of carbon microstructure and electrochemical performance, highlighting dominant ion-storage mechanisms in Li-ion and Na-ion systems as a function of structural characteristics.

|  | $S_{BET}$  $(\frac{m2}{g})$ | $I_{D}$/$I_{G}$ | $d_{002}$  (average d-spacing) | Conductivity | | LIB*  mA h g⁻¹, ICE, mA g⁻¹ | NIB*  mA h g⁻¹, ICE, mA g⁻¹ |
| --- | --- | --- | --- | --- | --- | --- | --- |
| CD-12C | 26.2 | 1.02 | 0.402 nm | 934 S·m⁻¹ | | 213.7, 44.6, 50 | 174.5, 58.5, 50 |
| CD-14C | 38.6 | 1.13 | 0.373 nm | 1.3 kS·m⁻¹ | | 180.4, 46.4, 50 | 192.2, 48.1, 50 |
| CD-16C | 31.2 | 1.21 | 0.36 nm | 1.6 kS·m⁻¹ | | 200, 60.5, 50 | 74.3, 41.6, 50 |
| Ion-storage mechanisms | | | | | | | |
|  | Li-ion system | | | | Na-ion system | | |
| CD-12C | Sloping Capacity (> 0.1 V).  Storage Mechanism: Adsorption.  Controlling factor: Defects. | | | | Sloping Capacity (> 0.1 V).  Storage Mechanism: Adsorption.  Controlling factor: Defects. | | |
|  | Plateau Capacity (< 0.1 V).  Storage Mechanism: Intercalation/filling.  Controlling factor: Micropore. | | | | Plateau Capacity (< 0.1 V).  Storage Mechanism: Intercalation/filling.  Controlling factor: Micropore. | | |
| CD-14C | Sloping Capacity (> 0.1 V).  Storage Mechanism: Adsorption.  Controlling factor: Defects. | | | | Sloping Capacity (> 0.1 V).  Storage Mechanism: Adsorption.  Controlling factor: Defects. | | |
|  | Plateau Capacity (< 0.1 V).  Storage Mechanism: Intercalation/filling.  Controlling factor: Mesopore, closed pore, and graphitic domain. | | | | Plateau Capacity (< 0.1 V).  Storage Mechanism: Intercalation/filling.  Controlling factor: Mesopore, closed pore, and graphitic domain. | | |
| CD-16C | Sloping Capacity (> 0.1 V).  Storage Mechanism: Adsorption.  Controlling factor: Defects. | | | | Sloping Capacity (> 0.1 V).  Storage Mechanism: Adsorption.  Controlling factor: Defects. | | |
|  | Plateau Capacity (< 0.1 V).  Storage Mechanism: Intercalation/filling.  Controlling factor: Mesopore, closed pore, and graphitic domain. | | | | Plateau Capacity (< 0.1 V).  Storage Mechanism: Filling.  Controlling factor: Mesopore and closed pore. | | |
| *Reversible capacity (mA h g⁻¹), ICE (%), Current density (mA g⁻¹) | | | | | | | |


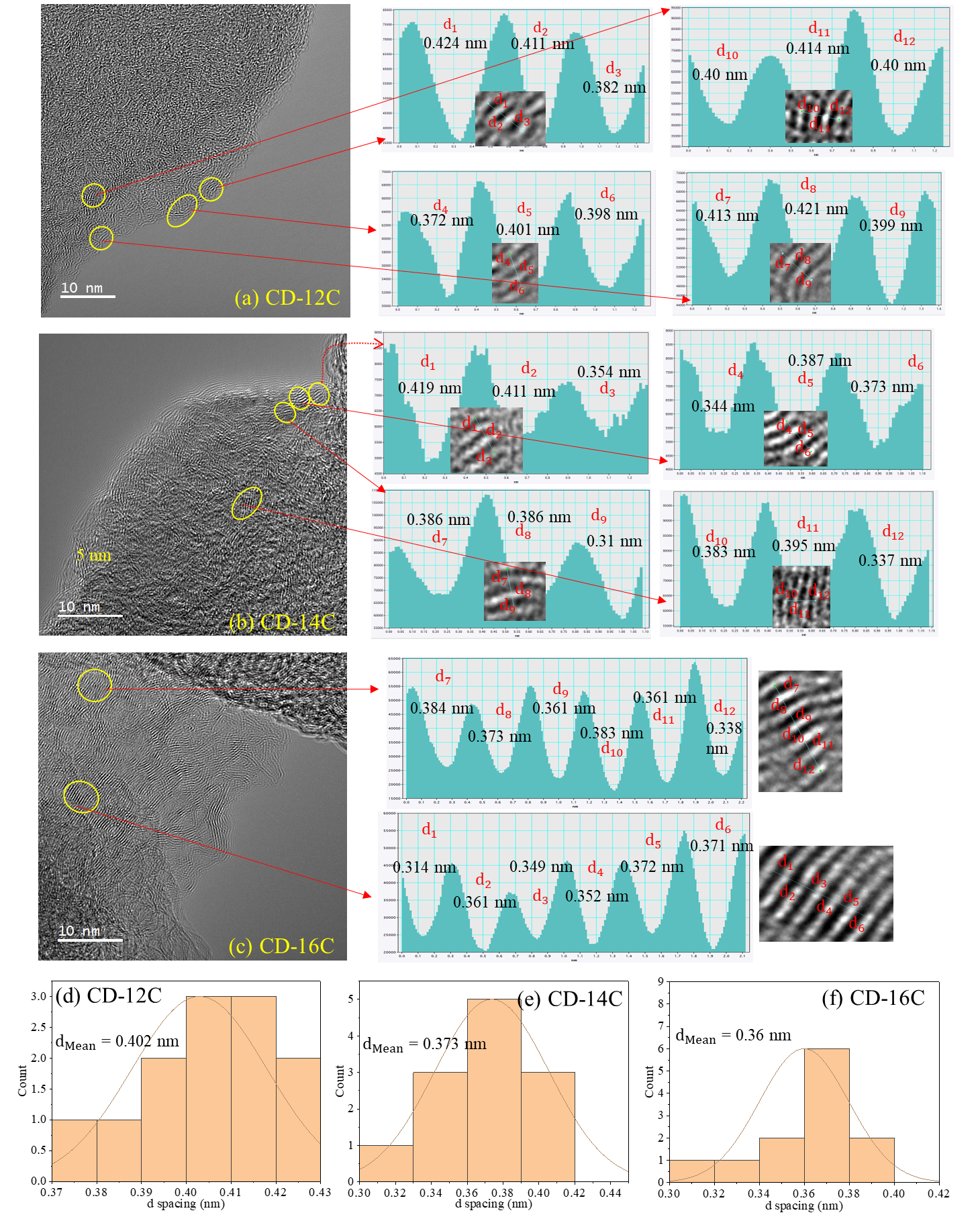


***Figure S1.*** *Average d-spacing of CD-12C (a), CD-14C (b), and CD-16C (c), along with corresponding histograms (d–f) showing d-spacing distributions measured at multiple locations.*


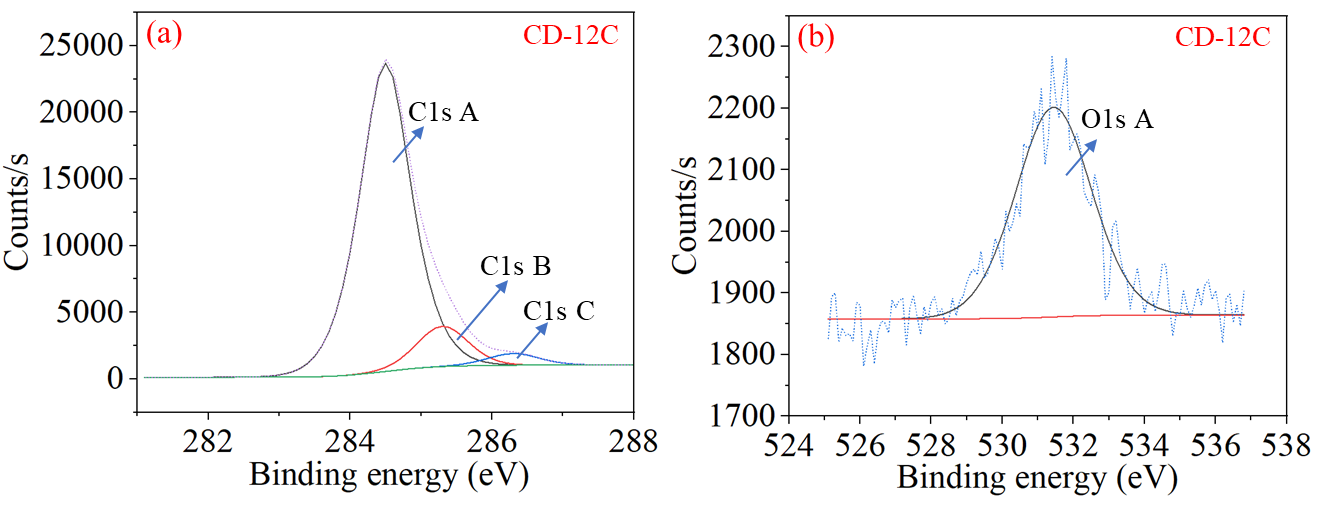


**Figure S2.** The XPS C1s (a) and O1s (b) deconvolution CD-12C.


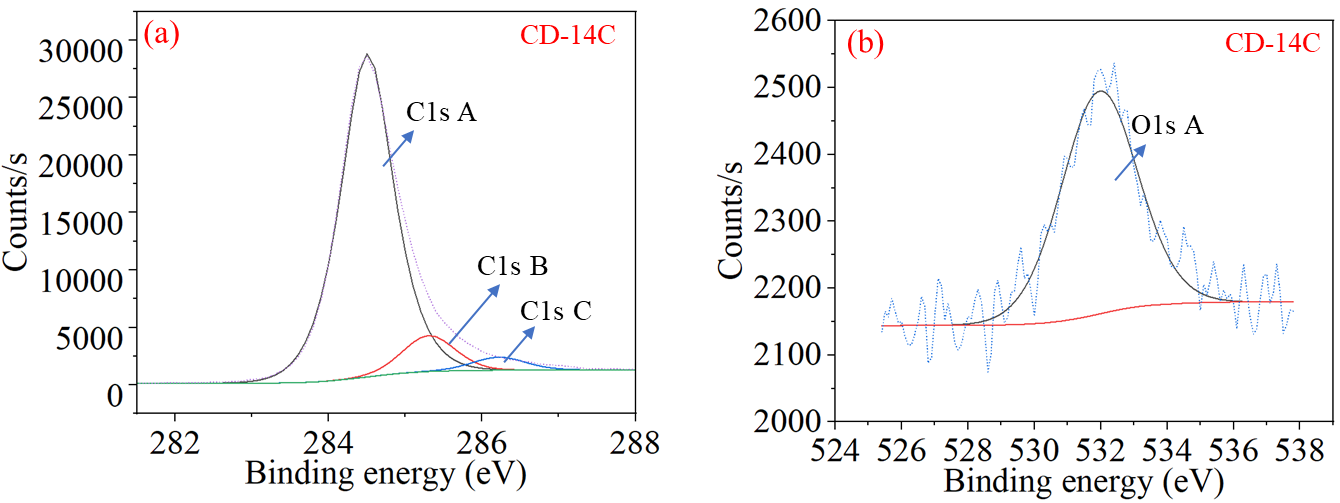


**Figure S3.** The XPS C1s (a) and O1s (b) deconvolution CD-14C.


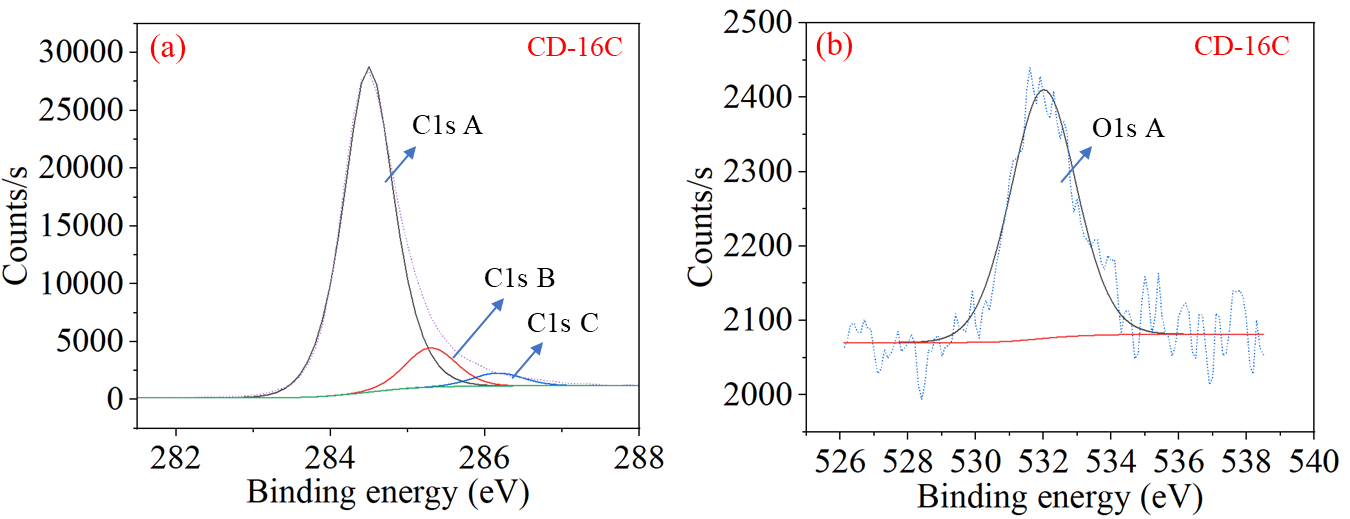


**Figure S4.** The XPS C1s (a) and O1s (b) deconvolution CD-16C.


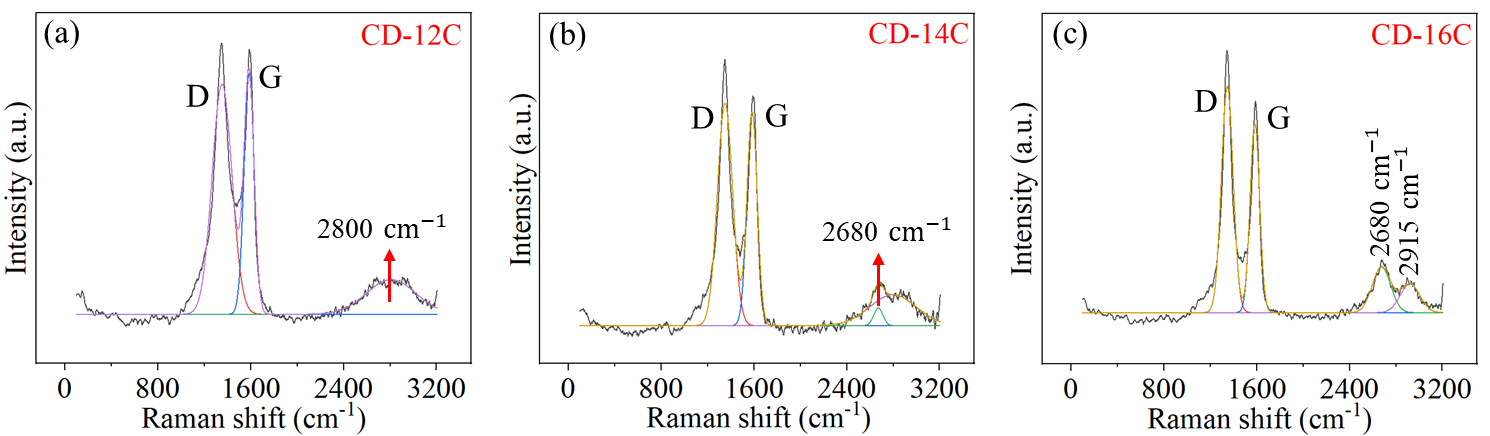


**Figure S5.** Raman spectra of all carbons; Fitted Raman spectra of a) CD-12C, b) CD-14C, and d) CD-16C using Gaussian line shape.

The Raman spectra of the reported carbons (**Fig. S5**) display characteristic D and G bands, which are typical of hybrid carbon matrices where ordered and disordered carbon domains coexist at the nanoscale. The D band, associated with structural defects and disorder in carbon sheets, shows a decrease in full width at half maximum (FWHM) as in-plane micro-ordering progresses through clustering of sp² carbon phases. This trend indicates that carbon–carbon bonding becomes more uniform and less defective with increased thermal treatment. As the degree of micro-ordering increases, both the D and G bands become narrower, reflecting enhanced structural organization. Additionally, the second-order 2D band—specific to graphitic structures—appears more prominently at a higher Raman shift (2682 cm⁻¹), particularly in the CD-16C sample. This sharpening and intensification of the 2D band further confirm a reduction in defects and an increase in the overall structural order of the carbon material [5].


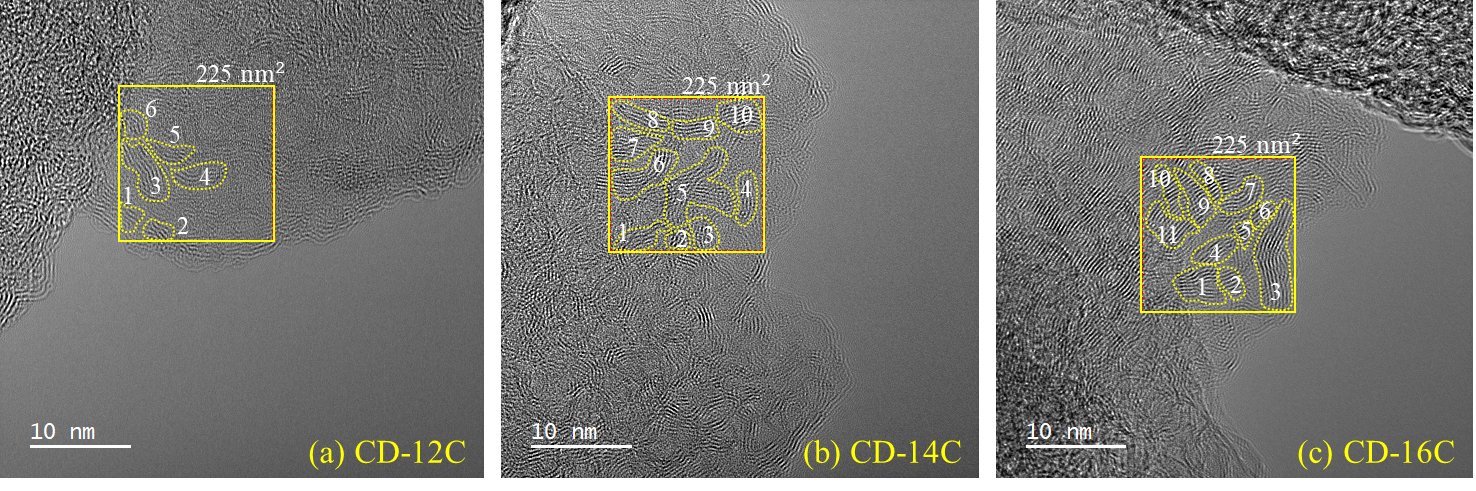


***Figure S6.*** *Approximate domain counts in CD-12C, CD-14C, and CD-16C.*


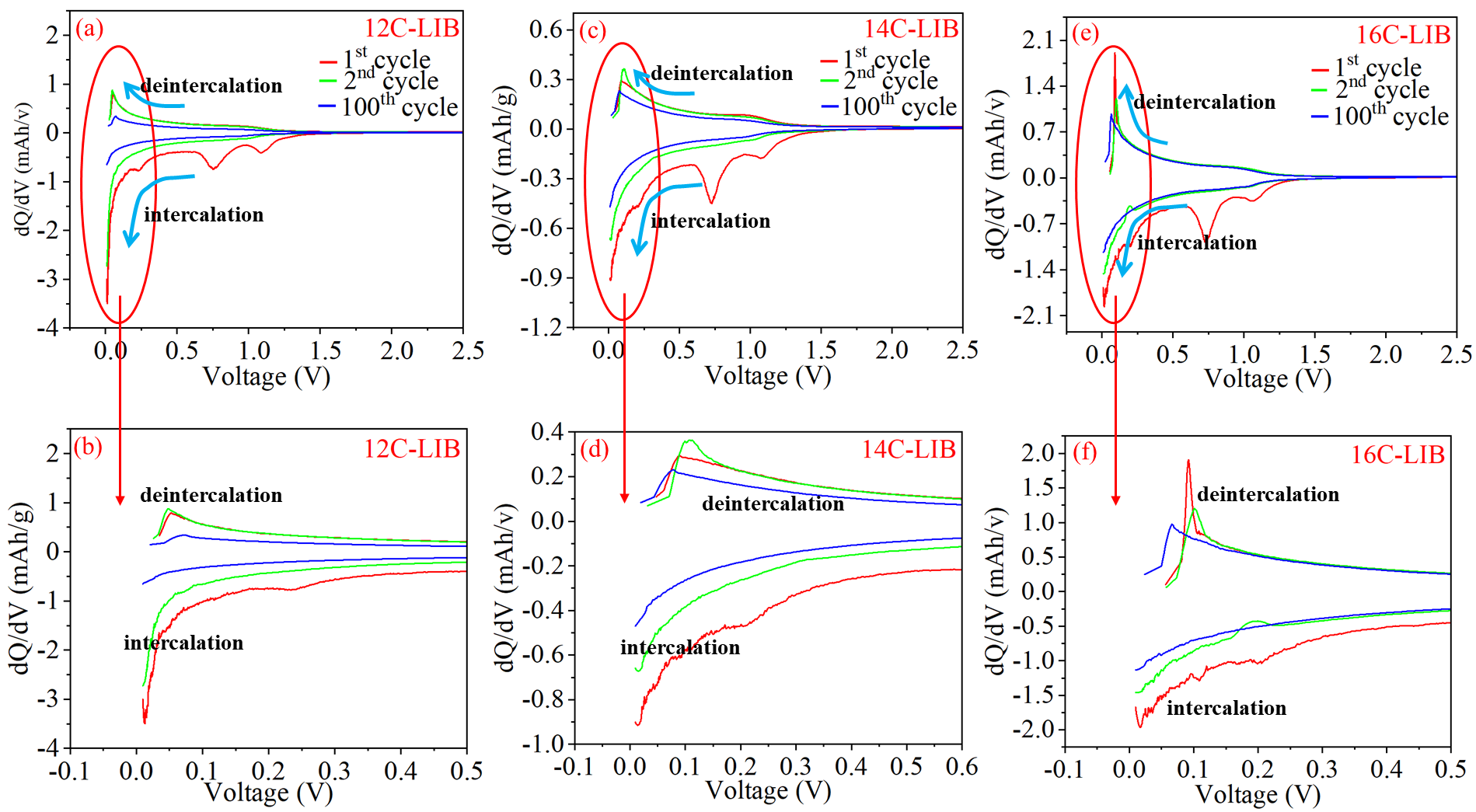


**Figure S7.** The illustration of dQ/dV curve of carbon anode from 1st, 2nd, and 100th in Li system, a, b) CD-12C-LIB, c, d) CD-14C-LIB, and e, f) CD-16C-LIB.

**Fig.  S7** presents the differential capacity curves of the electrodes during the 1st, 2nd, and 100th cycles in Li system. In the first lithiation scan, a prominent reduction peak appears around 0.8 V (vs. Li⁺/Li), which is attributed to the formation of the solid electrolyte interphase (SEI) layer. This process is typically associated with an irreversible electrochemical reaction in the anode. The disappearance of this reduction peak in subsequent lithiation cycles suggests that the initially formed SEI layer on the carbon is stable and effectively prevents further interaction between the electrolyte and the microstructured carbon domains. From the second cycle onward, the differential capacity plots (DCPs) remain consistent, indicating that the anodes reach a stable electrochemical state after the initial cycle. The lithiation (0.1–0.01 V) and delithiation (0.01–0.1 V) peaks observed in the DCPs are shown in **Figs. S7b, S7d,** and **S7f**, further illustrating the stable and reversible behavior of the electrodes over prolonged cycling.


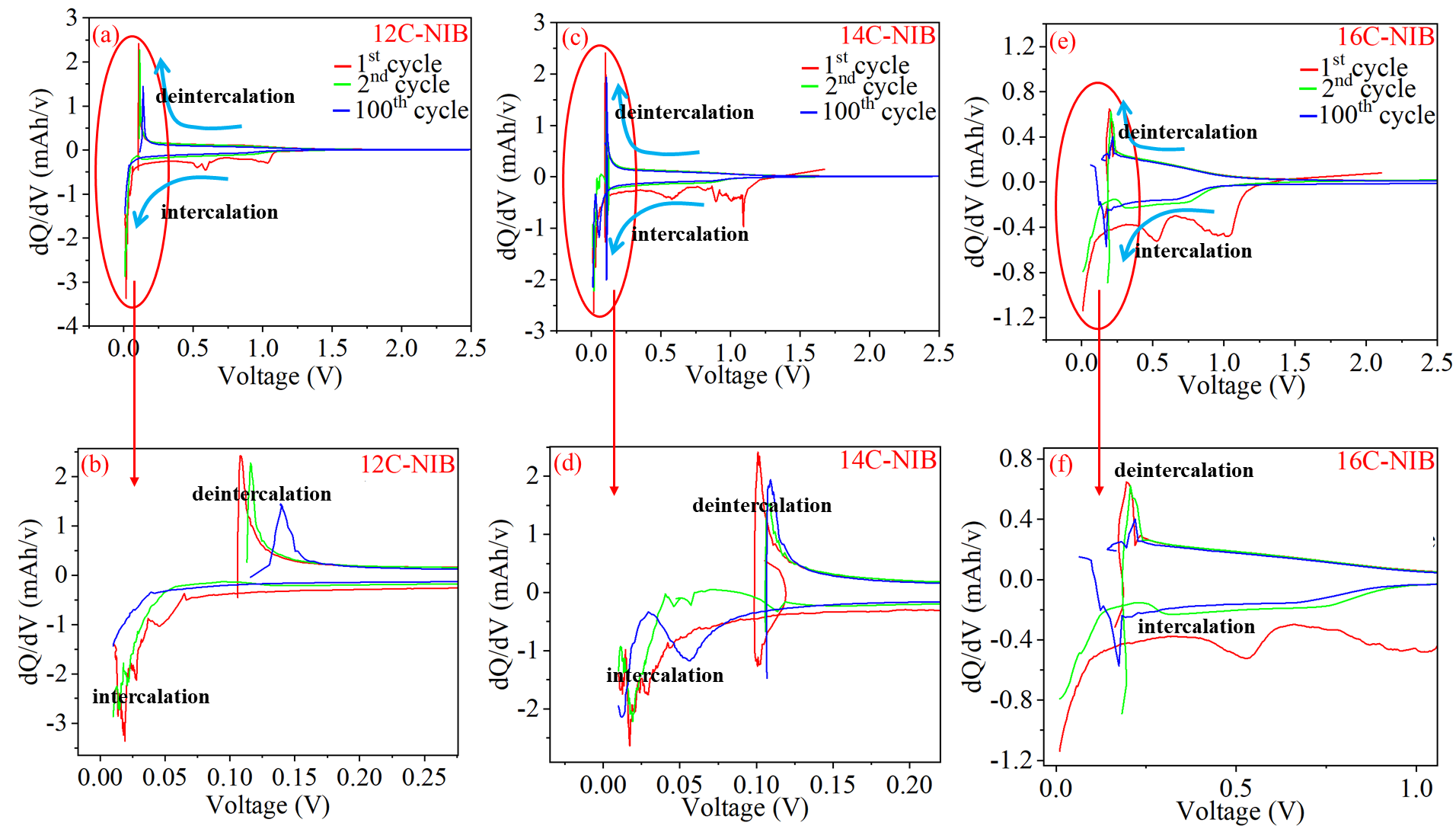


**Figure S8.** The illustration of dQ/dV curve of carbon anode from 1st, 2nd, and 100th in Na system, a, b) CD-12C-NIB, c, d) CD-14C-NIB, and e, f) CD-16C-NIB.

**Fig. S8** presents the differential capacity (dQ/dV) curves of the electrodes during the 1st, 2nd, and 100th cycles in Na system. In the first sodiation scan, a prominent reduction peak appears around 0.8 V (vs. Na⁺/Na), which is attributed to the formation of the solid electrolyte interphase (SEI) layer. This process is typically associated with an irreversible electrochemical reaction at the anode. The disappearance of this reduction peak in CD-12C-NIB and CD-14C-NIB during subsequent sodiation cycles suggests that the initially formed SEI layer on the carbon is stable and effectively prevents further interaction between the electrolyte and the microstructured carbon domains. From the second cycle onward, the differential capacity plots (DCPs) remain consistent, indicating that the anodes reach a stable electrochemical state after the initial cycle. However, the scenario is different for CD-16C-NIB, where a small overlapping peak reappears around 0.8 V in the 100th cycle. This suggests that the SEI layer formed in CD-16C-NIB is not stable and undergoes degradation and reformation during prolonged cycling. The sodiation (0.1–0.01 V) and desodiation (0.01–0.1 V) peaks observed in the DCPs, as shown in **Figs. S8b, S8d,** and **S8f**, further illustrate the stable and reversible electrochemical behavior of the electrodes over extended cycling.


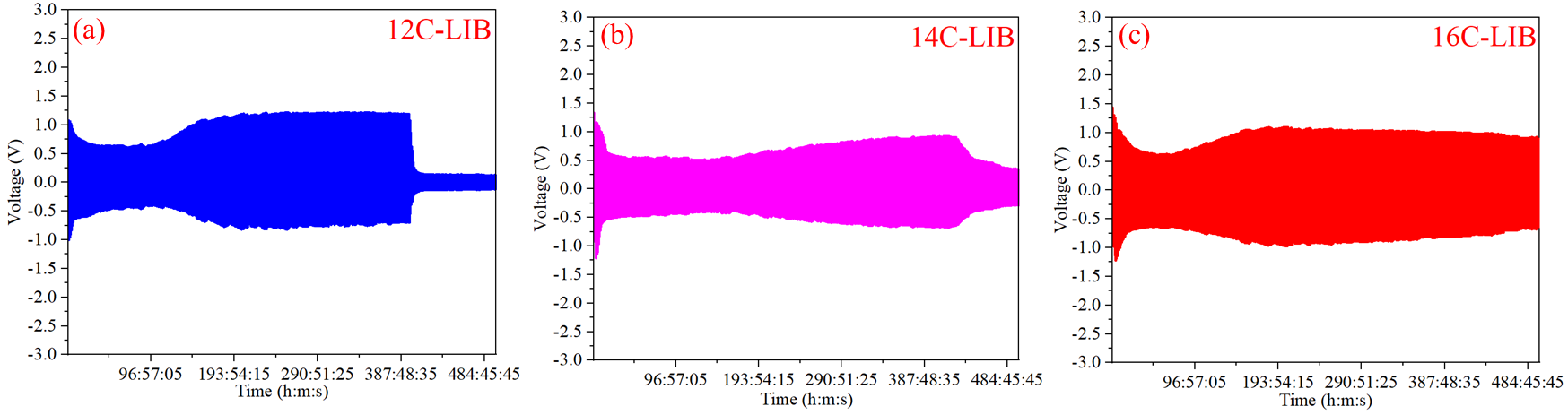


**Figure S9.** Galvanostatic cycling of a) CD-12C, b) CD-14C, and c) CD-16C symmetric cell in Li system.


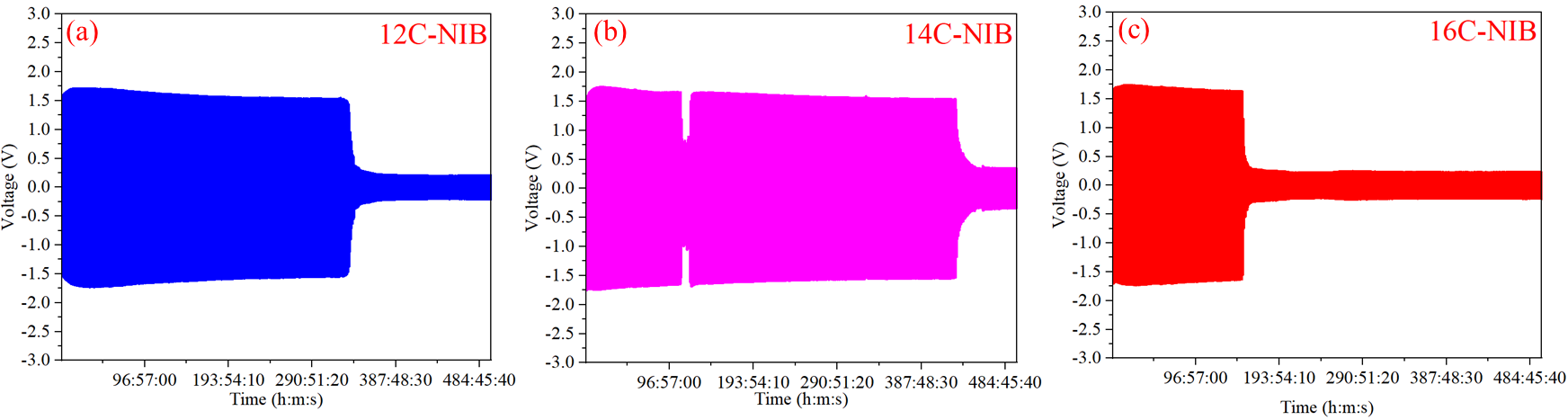


**Figure S10.** Galvanostatic cycling of a) CD-12C, b) CD-14C, and c) CD-16C symmetric cell in Na system.

As displayed in **Fig. S9**, the symmetric cells based on CD-16C-LIB in the lithium system exhibit relatively low voltage hysteresis and stable cycling performance for up to 500 hours. In contrast, CD-12C-LIB displays significant voltage fluctuations throughout the testing period, more so than CD-14C-LIB and CD-16C-LIB. A major short circuit was observed in CD-12C-LIB after approximately 400 hours, resulting in a permanent voltage drop. This disruption in the voltage profile is indicative of an internal short circuit within the CD-12C-LIB cell. On the other hand, both CD-14C-LIB and CD-16C-LIB show no signs of short circuits over the 500-hour cycling period, highlighting their superior electrochemical stability. In the sodium system, however, the behavior differs from that in the lithium system. As illustrated in **Fig. S10**, all cells initially demonstrate excellent cycling stability. Nevertheless, over extended cycling, each cell eventually experiences a permanent voltage drop. Among them, CD-14C-NIB performs the best. Although it initially undergoes a voltage drop—likely due to a short circuit—it recovers in subsequent cycles. However, around 440 hours, it suffers a severe short circuit, leading to a permanent voltage failure. Overall, the superior cycling stability of CD-16C-LIB compared to the other electrodes may be attributed to the formation of a robust and stable solid electrolyte interphase (SEI) layer. This SEI not only suppresses side reactions and stabilizes the electrode/electrolyte interface but also facilitates efficient Li⁺ ion diffusion through the layer, contributing to enhanced electrochemical performance [6].


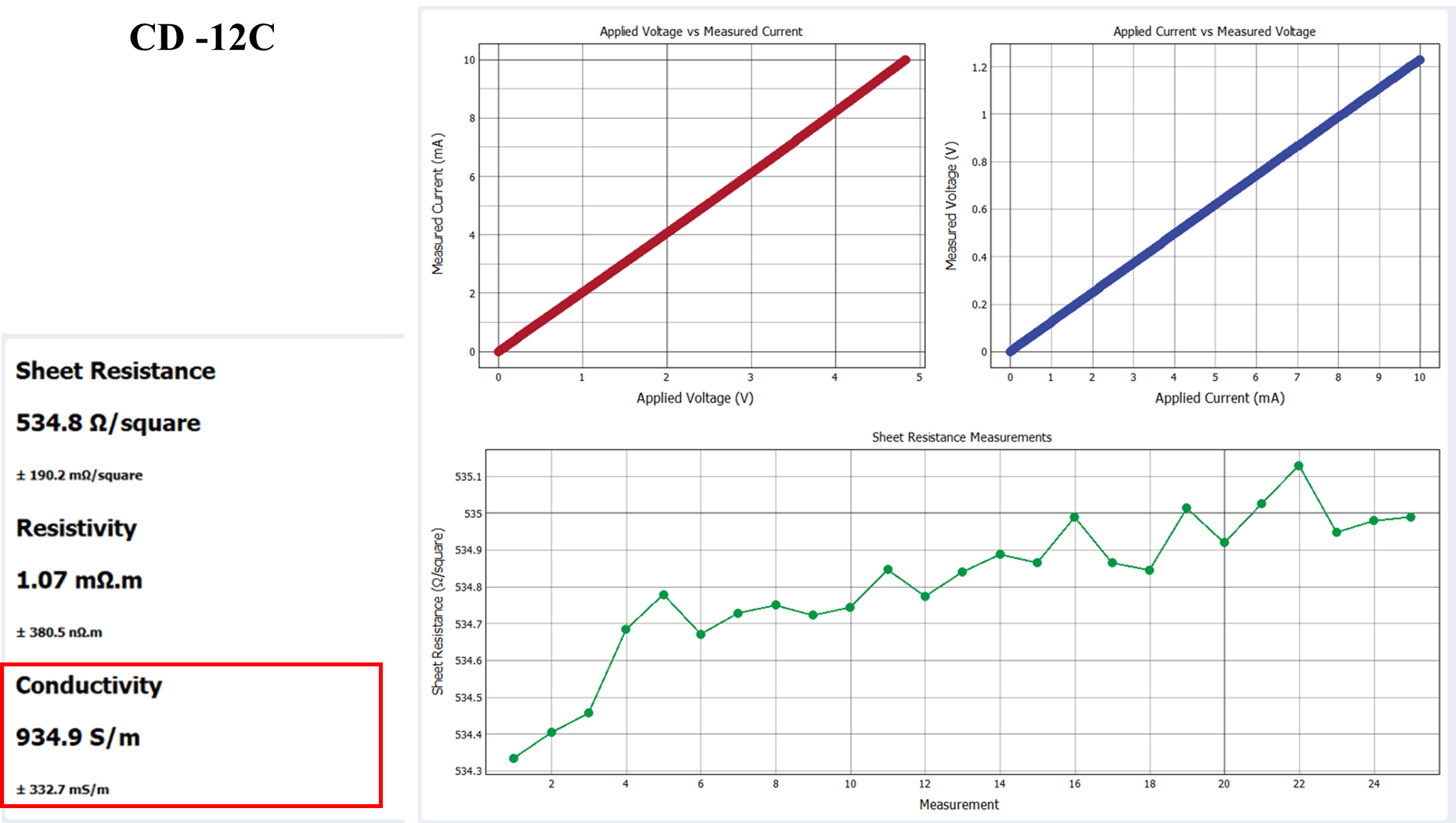


**Figure S11.** Conductivity of CD-12C measured using Ossila Four-Point Probe.


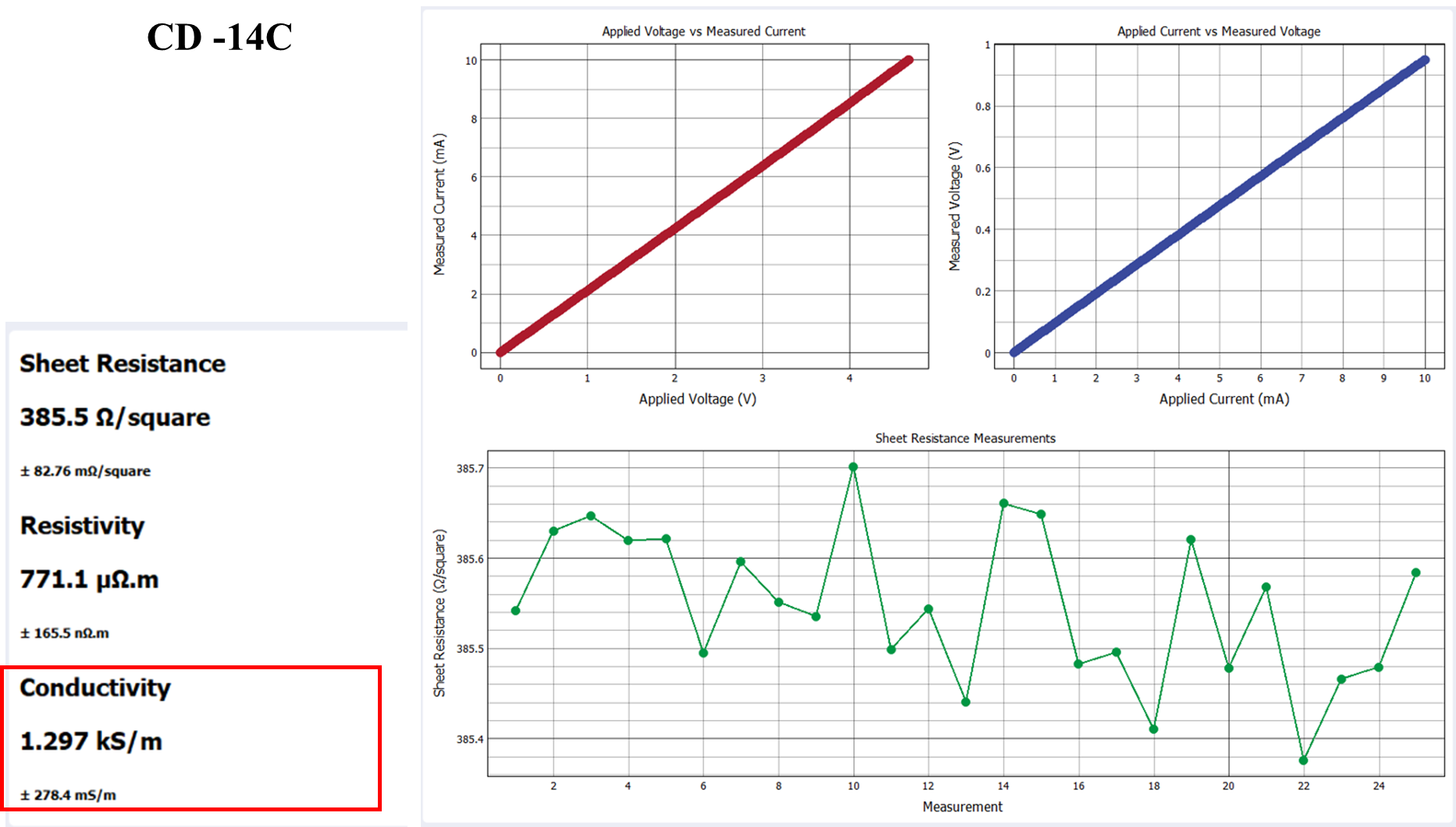


**Figure S12.** Conductivity of CD-14C measured using Ossila Four-Point Probe.


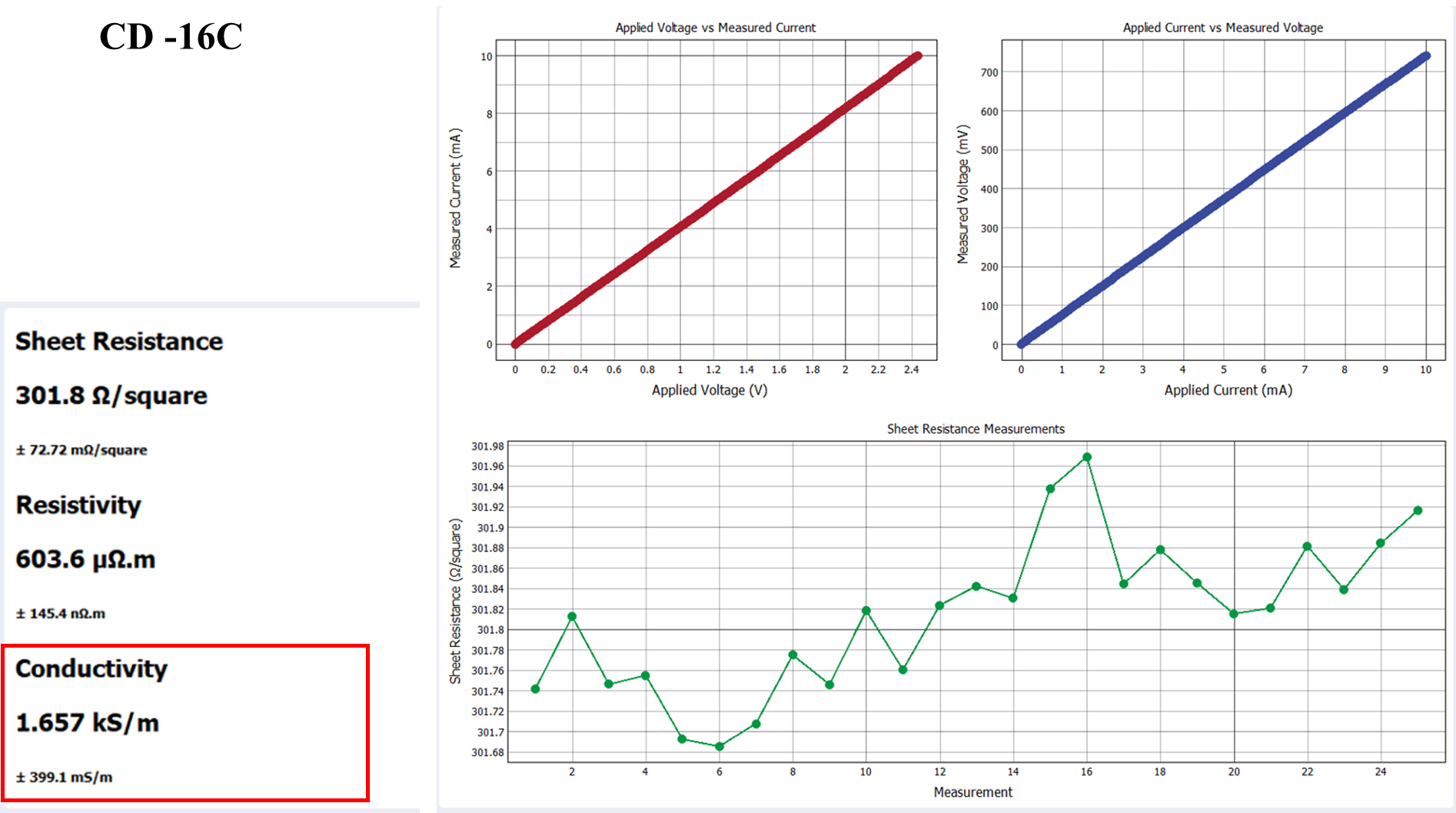


**Figure S13.** Conductivity of CD-16C measured using Ossila Four-Point Probe.


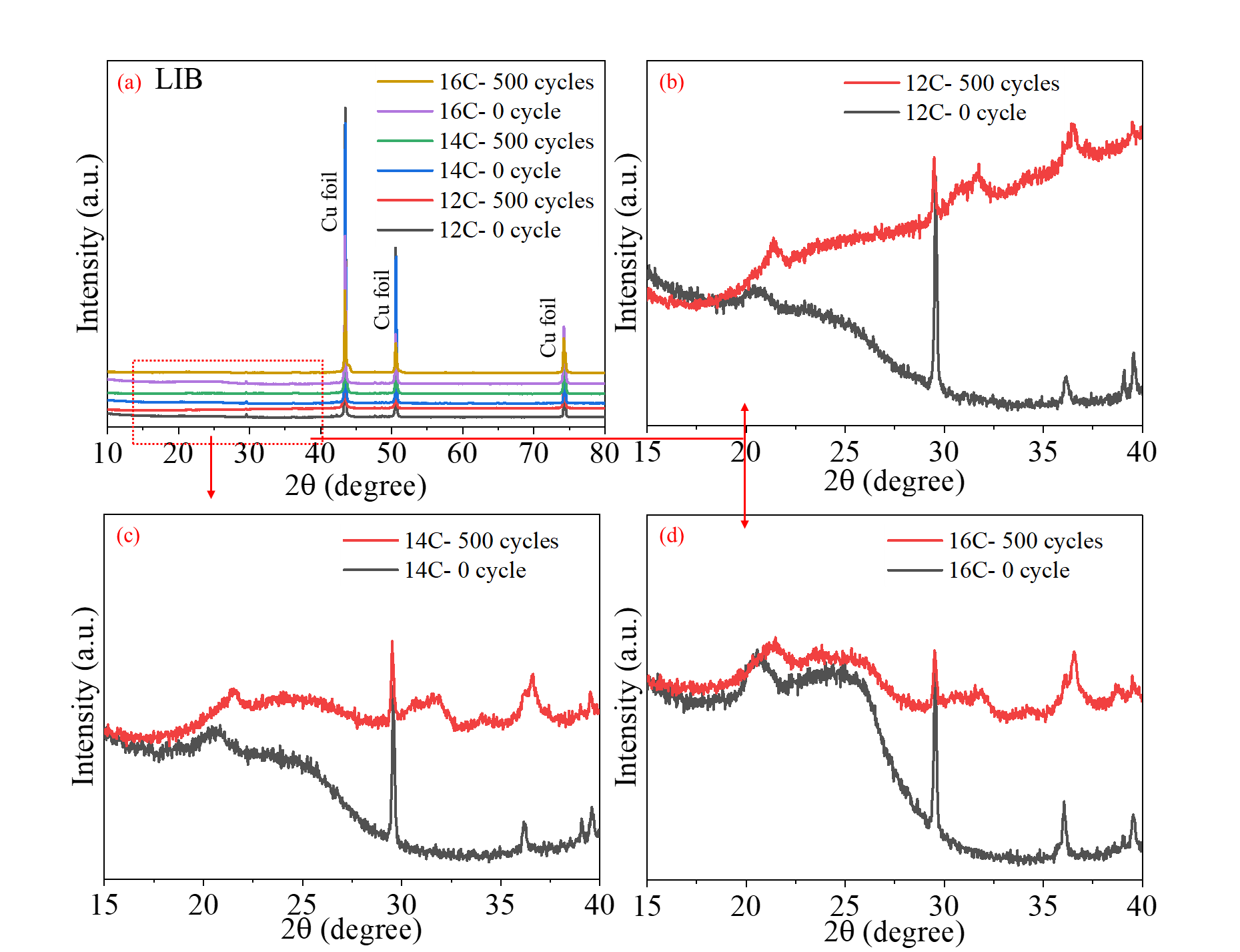


**Figure S14.** X-ray diffractogram of electrode materials before and after cycling (500 cycles at a current density of 500 mA·g⁻¹): (a) CD-12C-LIB, (b) CD-14C-LIB, and (c) CD-16C-LIB.


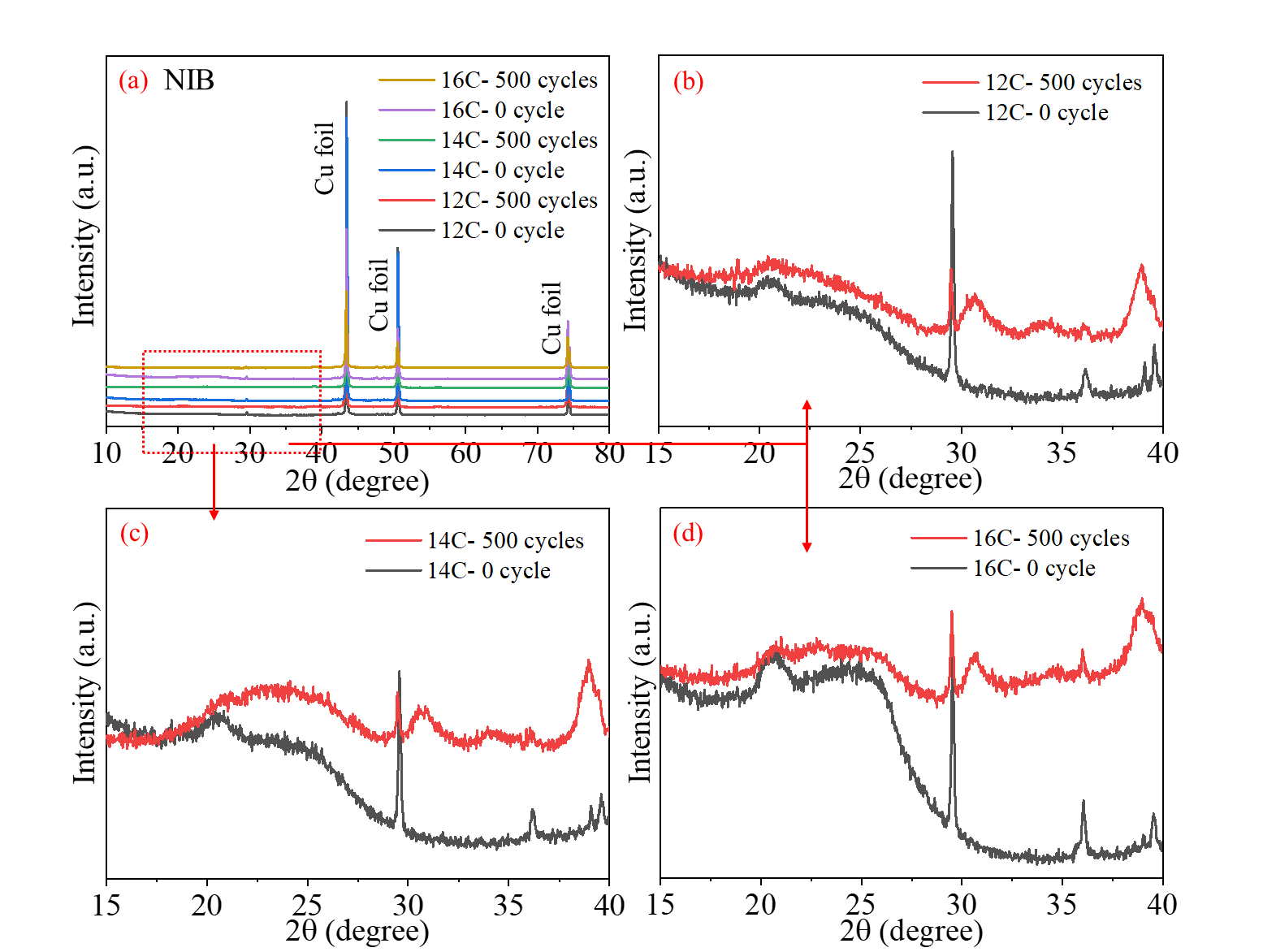


**Figure S15.** X-ray diffractogram of electrode materials before and after cycling (500 cycles at a current density of 500 mA·g⁻¹): (a) CD-12C-NIB, (b) CD-14C-NIB, and (c) CD-16C-NIB.


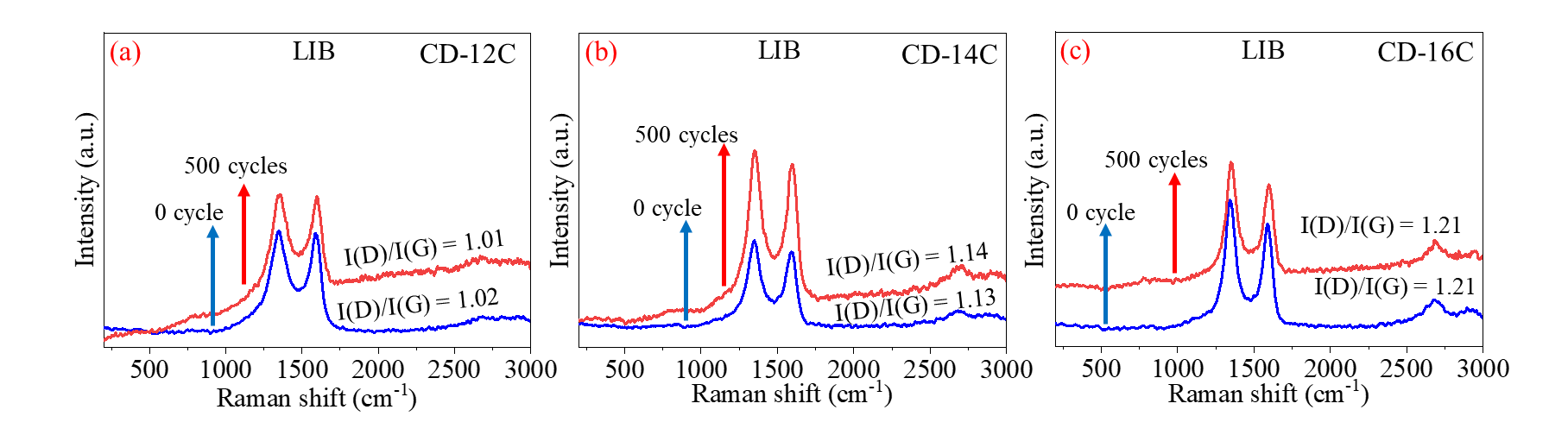


**Figure S16.** Raman spectra of electrode materials before and after cycling (500 cycles at a current density of 500 mA·g⁻¹): (a) CD-12C-LIB, (b) CD-14C-LIB, and (c) CD-16C-LIB.


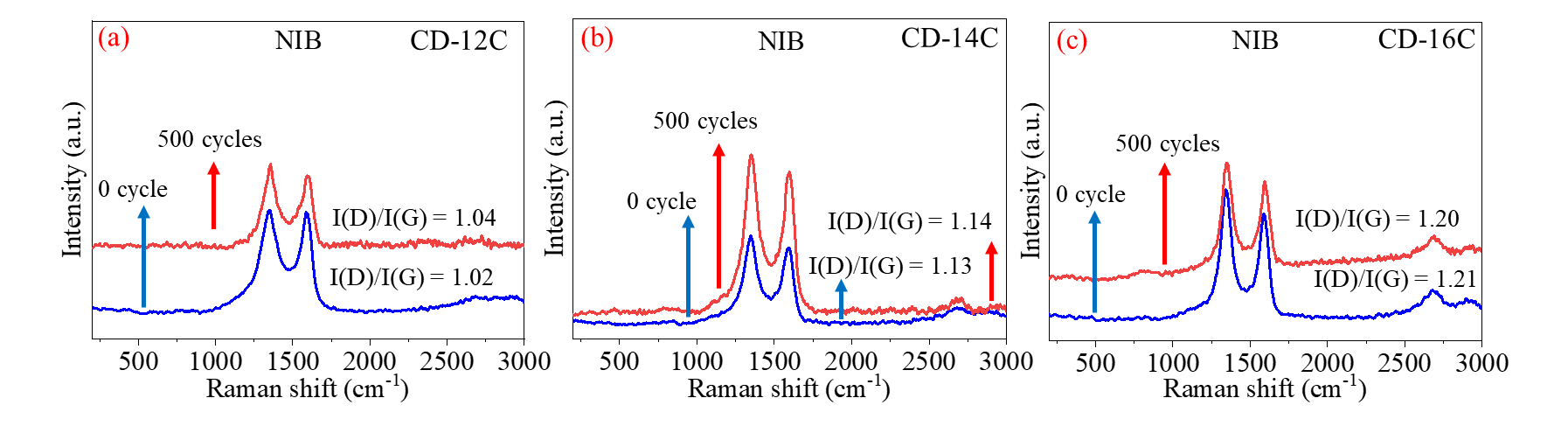


**Figure S17.** Raman spectra of electrode materials before and after cycling (500 cycles at a current density of 500 mA·g⁻¹): (a) CD-12C-NIB, (b) CD-14C-NIB, and (c) CD-16C-NIB.


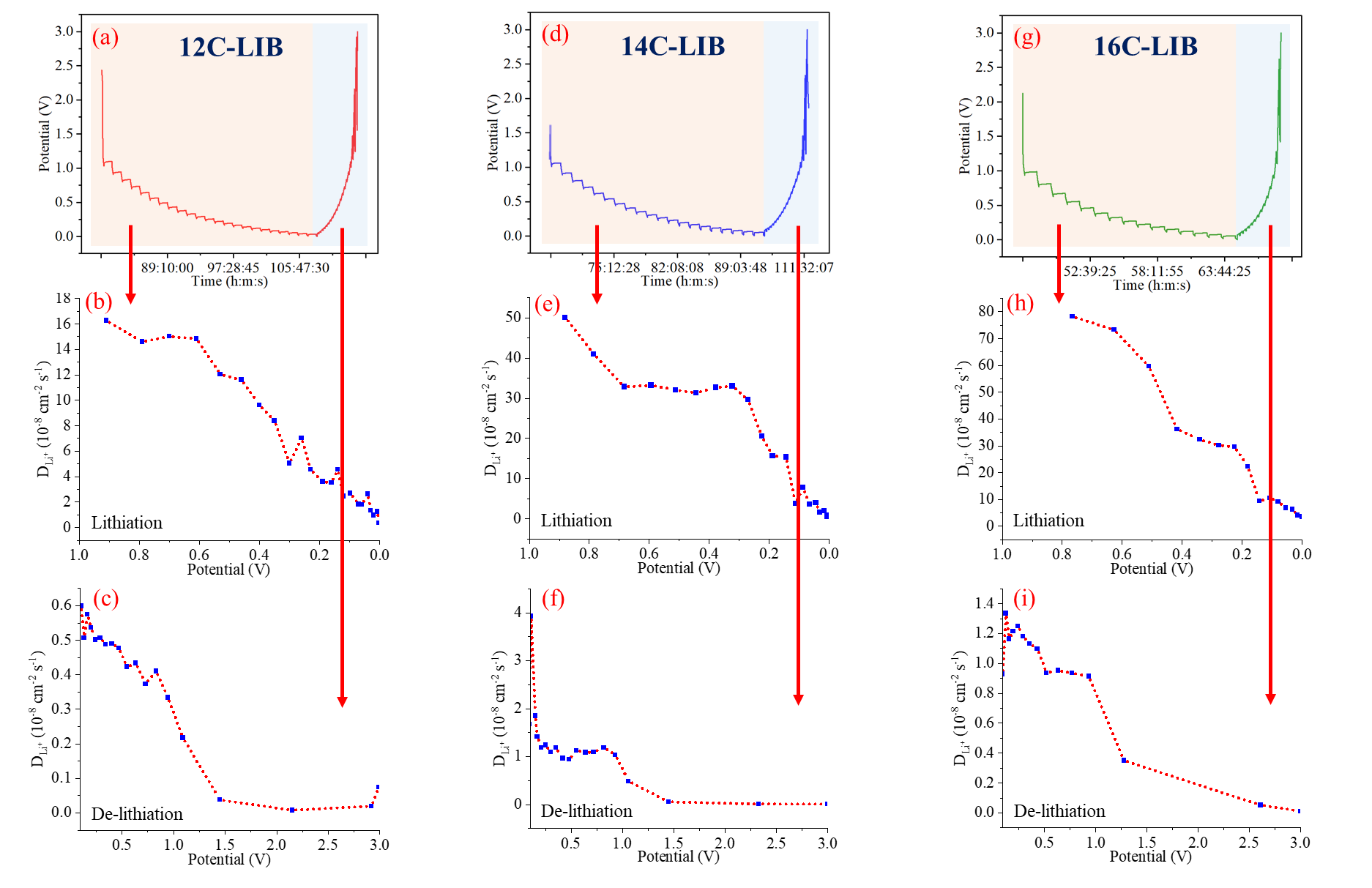


**Figure S18.** a) The galvanostatic intermittent titration technique (GITT) curves of CD-12C-LIB, b, c) and its calculated diffusivity coefficients 𝐷Li+ during lithiation and de-lithiation. d) The GITT curves of CD-14C-LIB, e, f) and its calculated diffusivity coefficients 𝐷Li+ during lithiation and de-lithiation. g) The GITT curves of CD-16C-LIB, h, i) and its calculated diffusivity coefficients 𝐷Li+ during lithiation and de-lithiation.


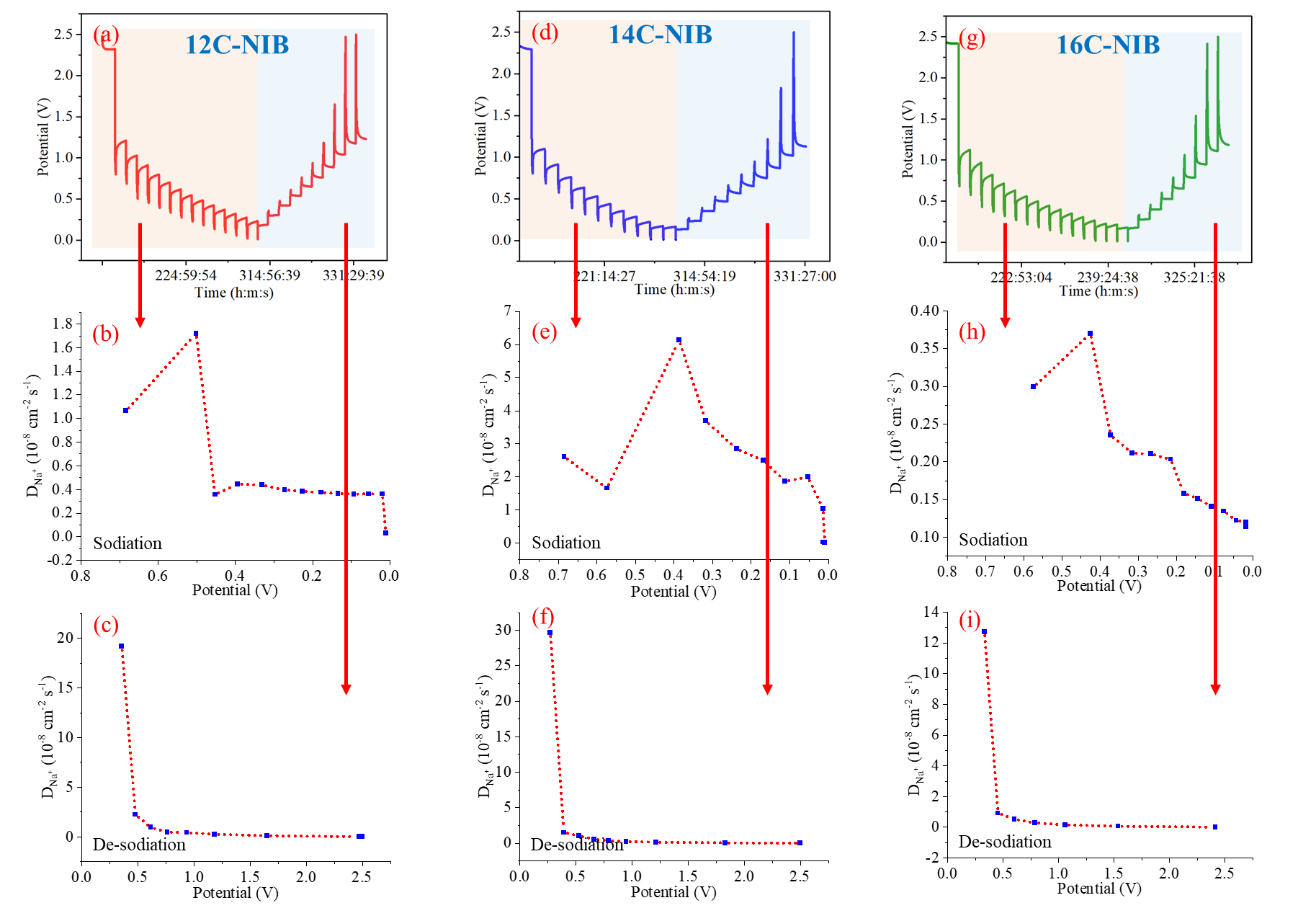


**Figure S19.** a) The galvanostatic intermittent titration technique (GITT) curves of CD-12C-NIB, b, c) and its calculated diffusivity coefficients 𝐷Na+ during sodiation and de-sodiation. d) The GITT curves of CD-14C-NIB, e, f) and its calculated diffusivity coefficients 𝐷Na+ during sodiation and de-sodiation. g) The GITT curves of CD-16C-NIB, h, i) and its calculated diffusivity coefficients 𝐷Na+ during sodiation and de-sodiation.

**Technoeconomic valuation of the carbonization process**

Waste CDs were processed into carbon using a single-step selective thermal transformation technique, a straightforward approach from a techno-economic perspective. Since carbonization is a thermal process, a 0.5-ton Rotary Carbonization Furnace rating 22 kW was modelled to simulate large-scale production, while a Horizontal Tube Furnace rating 6 kW was used for lab-scale synthesis. Cost calculations (**Tables S8-S11**) considered key consumables such as electricity and nitrogen, with raw material cost assumed to be zero as waste CDs have no market value. Baseline prices included electricity at 0.267–0.45 AUD per kWh and nitrogen at approximately 1 AUD per kilogram. Based on this analysis, the production cost of carbon from waste CDs is estimated at 76 – 165 AUD per gram at lab scale and approximately 6 – 10 AUD per kilogram at large scale.

**Table S8.** Electrical energy consumption for lab-scale experiments.

| Power consumption breakdown | | |
| --- | --- | --- |
| Horizontal Tube Furnace (rating: 6 kW) | | |
| **1200** $\boldsymbol{℃}$ | **1400** $\boldsymbol{℃}$ | **1600** $\boldsymbol{℃}$ |
| **Ramp up + hold:** (12 + 2) hrs at full power.  **Ramp down:** 6 hrs at half power.  $E_{\text{lab}}=6\text{ kW}\times14\text{ h}+6\text{ kW}\times0.5\times6\text{ h}=84+18=102\text{ kWh}$ | **Ramp up + hold:** (14 + 2) hrs at full power.  **Ramp down:** 7 hrs at half power.  $E_{\text{lab}}=6\text{ kW}\times16\text{ h}+6\text{ kW}\times0.5\times7 \text{h}=96+21=117\text{ kWh}$ | **Ramp up + hold:** (16 + 2) hrs at full power.  **Ramp down:** 8 hrs at half power.  $E_{\text{lab}}=6\text{ kW}\times18\text{ h}+6\text{ kW}\times0.5\times8 \text{h}=108+24=132\text{ kWh}$ |

**Table S9.** Comprehensive cost breakdown at laboratory scale carbonization.

|  | **1200C** | **1400C** | **1600C** |
| --- | --- | --- | --- |
| Power consumption | $\text{102 kWh}$ | $\text{117 kWh}$ | $\text{132 kWh}$ |
| Average Cost of the KWh is Australia 0.267 to 0.45 (AUD)/KWh | 27.32 – 45.9 AUD | 31.24 – 52.65 AUD | 35.24 – 59.4 AUD |
| Raw material (waste CDs) | N/A | N/A | N/A |
| 2 gm CD (18 % yield) | 0.36 gm | 0.36 gm | 0.36 gm |
| N_2_ gas (1 L/min) (for holding time; 2h; 0.2kg N_2_) | 0.2 AUD | 0.2 AUD | 0. 2 AUD |
| Total (AUD) for 0.36 gm carbon | 27.4 – 46.1 | 31.4 – 52.8 | 35.4 – 59.6 |
| **Cost per gm (AUD)** | 76.1 – 182.0 | 87.3 – 146.8 | 98.4 – 165.5 |

**Table S10.** Electrical energy consumption for large-scale experiments.

| Power consumption breakdown | | |
| --- | --- | --- |
| Carbonisation Furnace (rating: 22 kW) | | |
| **1200** $\boldsymbol{℃}$ | **1400** $\boldsymbol{℃}$ | **1600** $\boldsymbol{℃}$ |
| **Phase 1: Ramp up (**12 h)  Energy input, phase 1, $E_{\text{p1}}$: 22 kW × 12 h = **264 kWh**  **Phase 2: Carbonisation**  **Run – 1:**  Carbon produced: 50 kg  Hold: 2 h  E_R-1_: 22 kW × 2 h = **44 kWh**  **Run – 2:**  Carbon produced: 50 kg  Hold: 2 h  E_R-2_: 22 kW × 2 h = **44 kWh**  **Run – 3:**  Carbon produced: 50 kg  Hold: 2 h  E_R-3_: 22 kW × 2 h = **44 kWh**  Energy input, phase 2, $E_{\text{p2}}$: E_R-1_ + E_R-2_ + E_R-3_ = **132 kWh**  **Phase 3: Ramp down** (6 h)  Energy input, phase 3, $E_{\text{p3}}$: 22 kW × 6 h = **132 kWh**  **Total Energy Input =** $\boldsymbol{E}_{\text{p1}}$ **+** $\boldsymbol{E}_{\text{p2}}$ **+** $\boldsymbol{E}_{\text{p3}}$ **= 528 kWh** | **Phase 1: Ramp up (**14 h)  Energy input, phase 1, $E_{\text{p1}}$: 22 kW × 14 h = **308 kWh**  **Phase 2: Carbonisation**  **Run – 1:**  Carbon produced: 50 kg  Hold: 2 h  E_R-1_: 22 kW × 2 h = **44 kWh**  **Run – 2:**  Carbon produced: 50 kg  Hold: 2 h  E_R-2_: 22 kW × 2 h = **44 kWh**  **Run – 3:**  Carbon produced: 50 kg  Hold: 2 h  E_R-3_: 22 kW × 2 h = **44 kWh**  Energy input, phase 2, $E_{\text{p2}}$: E_R-1_ + E_R-2_ + E_R-3_ = **132 kWh**  **Phase 3: Ramp down** (7 h)  Energy input, phase 3, $E_{\text{p3}}$: 22 kW × 7 h = **154 kWh**  **Total Energy Input =** $\boldsymbol{E}_{\text{p1}}$ **+** $\boldsymbol{E}_{\text{p2}}$ **+** $\boldsymbol{E}_{\text{p3}}$ **= 594 kWh** | **Phase 1: Ramp up (**16 h)  Energy input, phase 1, $E_{\text{p1}}$: 22 kW × 16 h = **352 kWh**  **Phase 2: Carbonisation**  **Run – 1:**  Carbon produced: 50 kg  Hold: 2 h  E_R-1_: 22 kW × 2 h = **44 kWh**  **Run – 2:**  Carbon produced: 50 kg  Hold: 2 h  E_R-2_: 22 kW × 2 h = **44 kWh**  **Run – 3:**  Carbon produced: 50 kg  Hold: 2 h  E_R-3_: 22 kW × 2 h = **44 kWh**  Energy input, phase 2, $E_{\text{p2}}$: E_R-1_ + E_R-2_ + E_R-3_ = **132 kWh**  **Phase 3: Ramp down** (8 h)  Energy input, phase 3, $E_{\text{p3}}$: 22 kW × 8 h = **176 kWh**  **Total Energy Input =** $\boldsymbol{E}_{\text{p1}}$ **+** $\boldsymbol{E}_{\text{p2}}$ **+** $\boldsymbol{E}_{\text{p3}}$ **= 660 kWh** |

**Table S11.** Comprehensive cost breakdown at large scale carbonization.

|  | **1200C** | **1400C** | **1600C** |
| --- | --- | --- | --- |
| Power consumption | $\text{528 kWh}$ | $\text{594 kWh}$ | $\text{660 kWh}$ |
| Average Cost of the KWh is Australia 0.267 to 0.45 (AUD)/KWh | 140.9 – 237.6 AUD | 158.6 – 267.3 AUD | 176.2 – 297.0 AUD |
| Raw material (waste CDs) | N/A | N/A | N/A |
| Total carbon produced | 150 kg | 150 kg | 150 kg |
| N_2_ gas (200 L/min) (for holding time; phase 2; 90 kg N_2_) | 90 AUD | 90 AUD | 90 AUD |
| Total (AUD) for 150 kg carbon | 230.9 – 327.6 | 248.6 – 357.3 | 266.2 – 387.0 |
| **Cost per kg (AUD)** | 1.54 – 2.18 | 1.66 – 2.38 | 1.77 – 2.58 |
| *Allowing for typical industrial overheads (labour, maintenance, and depreciation), the cost per kg (AUD) is multiplied by 4. | | | |
| **Total carbonisation cost (AUD)** | 6.16 – 8.72 | 6.64 – 9.52 | 7.08 – 10.32 |

**LCA of the carbonization process**

This study explores the electrochemical properties of various carbon materials synthesized from waste compact discs (CDs). To complement the experimental findings, a comprehensive Life Cycle Assessment (LCA) has been conducted to quantify the environmental footprint associated with the carbonization process.

**Goal and scope of the LCA**

The LCA model developed in this study adheres to the standards outlined in ISO 14044:2006 – Environmental Management: Life Cycle Assessment – Requirements and Guidelines. The assessment was performed using **SimaPro 9.6.0.1**, a widely recognized LCA software tool. The primary goal of this LCA is to quantitatively evaluate the environmental impacts, both beneficial and adverse of carbonizing waste CDs into carbon at two different scales: laboratory scale and industrial scale-up. The carbonization process flow is illustrated in **Fig. S20**. A total of 19 impact categories were considered in this assessment, providing a detailed and multidimensional view of the environmental implications across various domains such as human health, climate change, resource depletion, and ecosystem quality.


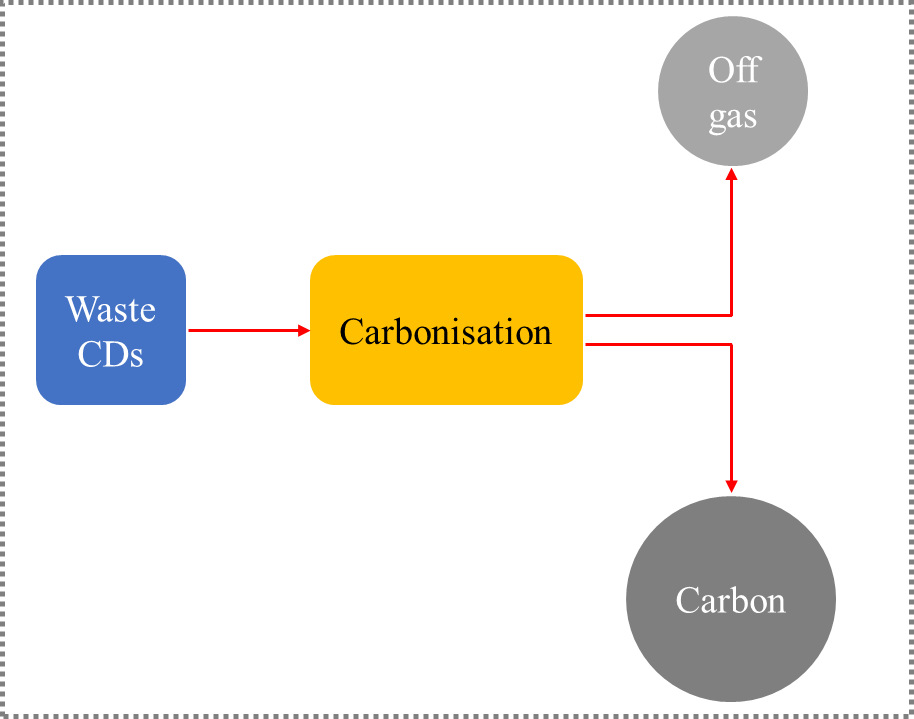


**Figure S20.** System boundary for carbonization.

**Life cycle inventory (LCI) analysis**

The material materials utilized in this study are summarized in **Table S12**. The carbonization process was conducted in a single-step procedure, without the use of any additional chemicals, ensuring a straightforward and clean conversion of waste CDs into carbon. The LCI has been categorized into two distinct operational scales: (i) Laboratory Scale and (ii) Small Industrial Scale-Up. For the scale-up scenario, a Rotary Continuous Carbonization Furnace with an energy consumption of 22 kWh was modelled to simulate large-scale production. Emissions generated during the carbonization process are detailed in **Table S12**, providing insight into the environmental outputs associated with each scale.

**Table S12.** Life cycle inventory result for carbonization of waste CDs (lab scale vs scale up study)

| **Input from Technosphere (material flow)** | | | | | | | | | | | | | |
| --- | --- | --- | --- | --- | --- | --- | --- | --- | --- | --- | --- | --- | --- |
|  | | Lab scale | | | | | | | Scale up | | | | |
| In-put per batch | | 2. 0 gm of raw material | | | | | | | 278 kg | | | | |
| Carier gas | | Argon | | | | | | | Argon/N_2_ | | | | |
|  | | In-put | | Residue | | Off gas | | | Total input | | Residue | Off gas | |
| Process breakdown | | 2.0 gm | | 0.36 – 0.4 gm | | - 1. - 1.6 gm | | | 834 kg | | 150 – 167 kg | 684 – 667 kg | |
| *The process yield is 18-20%. LCA has conducted using the pessimistic yield value of 18%. | | | | | | | | | | | | | |
| **Electricity (kWh)** | | | | | | | | | | | | | |
| Lab Scale | | | | | | | Scale up | | | | | | |
| Rating: 6 kW | | | | | | | Rating: 22 kW | | | | | | |
| Total Consumption:   1. 1200C – 102 kWh 2. 1400C – 117 kWh 3. 1600C – 132 kWh | | | | | | | Total Consumption:   1. 1200C – 528 kWh 2. 1400C – 594 kWh 3. 1600C – 660 kWh | | | | | | |
| **Emissions to air** | | | | | | | | | | | | | |
|  | 1200 $℃$ | | | | 1400 $℃$ | | | | | 1600 $℃$ | | | |
|  | Lab  (gm) | | Scale up (kg) | | Lab  (gm) | | | Scale up (kg) | | Lab  (gm) | | | Scale up (kg) |
| CO | 0.74948 | | 319.9 | | 0.80196 | | | 342.3 | | 0.84296 | | | 359.8 |
| CO_2_ | 0.30996 | | 132.3 | | 0.25912 | | | 110.6 | | 0.20172 | | | 86.1 |
| NO_2_ | 0.19844 | | 84.7 | | 0.22304 | | | 95.2 | | 0.25748 | | | 109.9 |
| H_2_ | 0.18696 | | 79.8 | | 0.21648 | | | 92.2 | | 0.23124 | | | 98.7 |
| O_2_ | 0.1394 | | 59.5 | | 0.10332 | | | 44.1 | | 0.08364 | | | 35.7 |
| CH_4_ | 0.010824 | | 4.62 | | 0.006888 | | | 2.94 | | 0.003444 | | | 1.47 |
| C₂H₄ | 0.002788 | | 1.19 | | - | | | - | | - | | | - |
| C₂H₂ | 0.002296 | | 0.98 | | - | | | - | | - | | | - |

**Environmental impact assessment**

To evaluate the environmental impacts of the process, 19 categories has been considered, the categories assessed in this study include Global warming (kg CO2 eq), Stratospheric ozone depletion (kg CFC11 eq), Ionizing radiation (kBq Co-60 eq), Ozone formation & Human health (kg NOx eq), Fine particulate matter formation (kg PM2.5 eq), Ozone formation, & Terrestrial ecosystems (kg NOx eq), Terrestrial acidification (kg SO2 eq), Freshwater eutrophication (kg P eq), Marine eutrophication (kg N eq), Terrestrial ecotoxicity (kg 1,4-DCB), Freshwater ecotoxicity (kg 1,4-DCB), Marine ecotoxicity (kg 1,4-DCB), Human carcinogenic toxicity (kg 1,4-DCB), Human non-carcinogenic toxicity (kg 1,4-DCB), Land use (m2a crop eq), Mineral resource scarcity (kg Cu eq), Fossil resource scarcity (kg oil eq), Water consumption (m3). The impact categories and their derived values have been summarized in **Table S13**.

**Figs. S21–S23** present a comparative impact assessment of the carbonization process for both lab-scale and large-scale carbon production across three temperatures: 1200 °C, 1400 °C, and 1600 °C. In the lab-scale scenario, electricity consumption emerges as the dominant contributor to environmental impacts across all categories. Emissions directly from the carbonization process itself have a minimal effect on the overall impact of values. Conversely, in the scale-up scenario, the trend reverses. Here, the carbonization process emissions contribute more significantly to the environmental burden than electricity consumption. However, when comparing the total impact values between lab-scale and large-scale processes, a drastic reduction is observed in the large-scale scenario. This reduction underscores the environmental suitability of scaling up the process, despite a slight increase in process-related emissions. This contrast becomes even more evident in the damage assessment figures (**Figs. S21b, S21d; S22b, S22d; S23b, S23d**), which evaluate impacts on resources, human health, and ecosystems. These figures clearly show that scaling up the process leads to a substantial decrease in numerical damage values across all categories.

**Fig. S24** provides a consolidated overview of the comparison. In **Fig.** **S24a**, each impact category for large-scale carbonization is shown to contribute significantly less than its lab-scale counterpart, with the large-scale contributions highlighted by dotted black boxes. **Figures S24b–S24c** further reinforce this by showing side-by-side comparisons of damage to resources, human health, and ecosystems—again confirming the lower environmental footprint of the large-scale method. One of the most critical insights from this life cycle assessment (LCA) is illustrated in **Figures S24d–S24e**, which focus on global warming potential. The lab-scale carbonization process contributes approximately 163815.3–211993.4 kg CO₂-equivalent per kg of carbon produced from waste CDs. In stark contrast, the scale-up process contributes only 3.94–5.5 kg CO₂-equivalent per kg of carbon, representing a massive reduction in greenhouse gas emissions. Finally, **Table S13** summarizes the total impacts for each carbonization process, further validating the environmental advantages of scaling up.


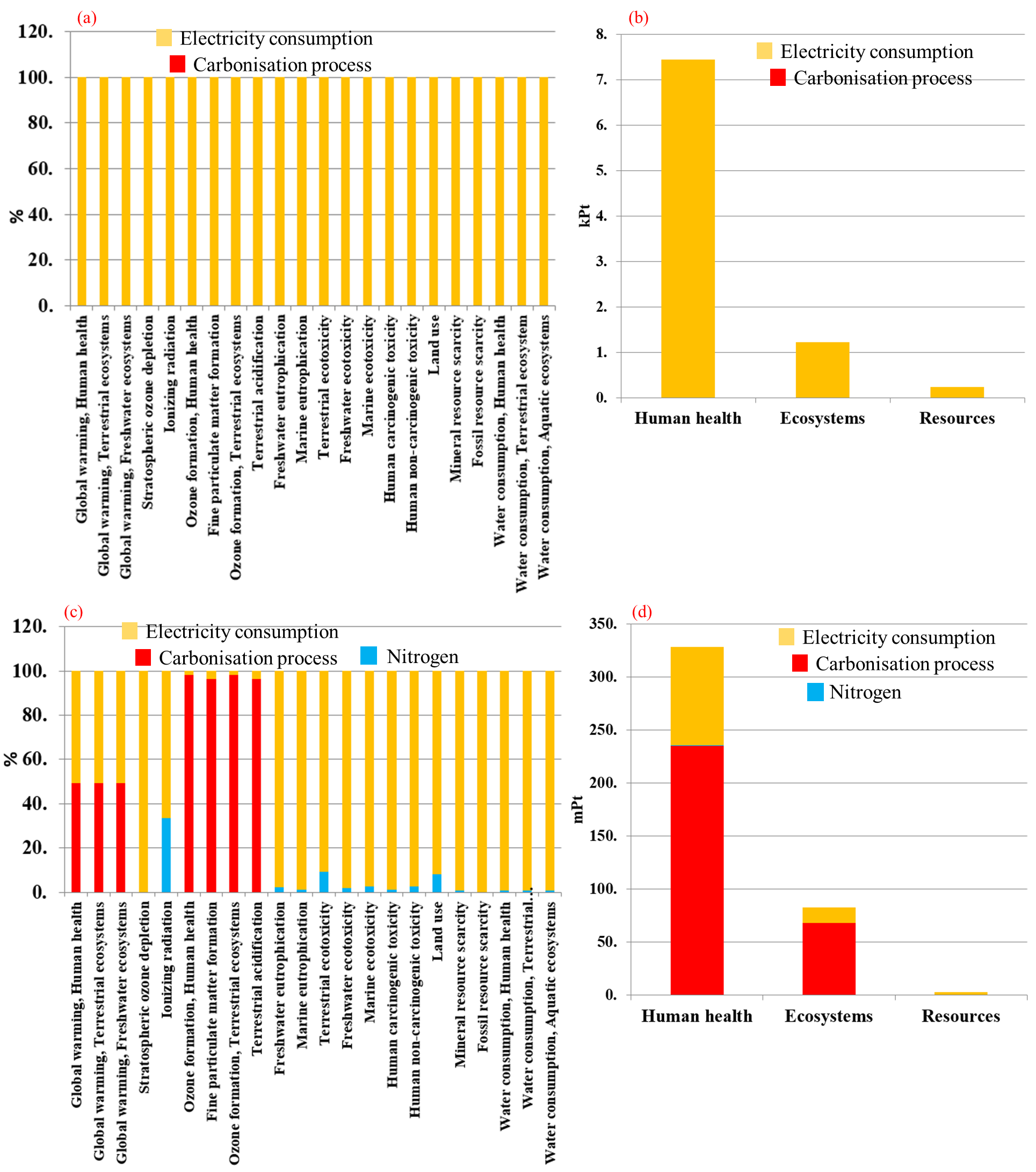


**Figure S21.** (a) Environmental impact profile of the carbonization process conducted at 1200 °C on a laboratory scale; (b) Damage assessment associated with the lab-scale carbonization process; (c) Environmental impact profile of the carbonization process at 1200 °C scaled to industrial levels; (d) Damage assessment of the large-scale carbonization process.


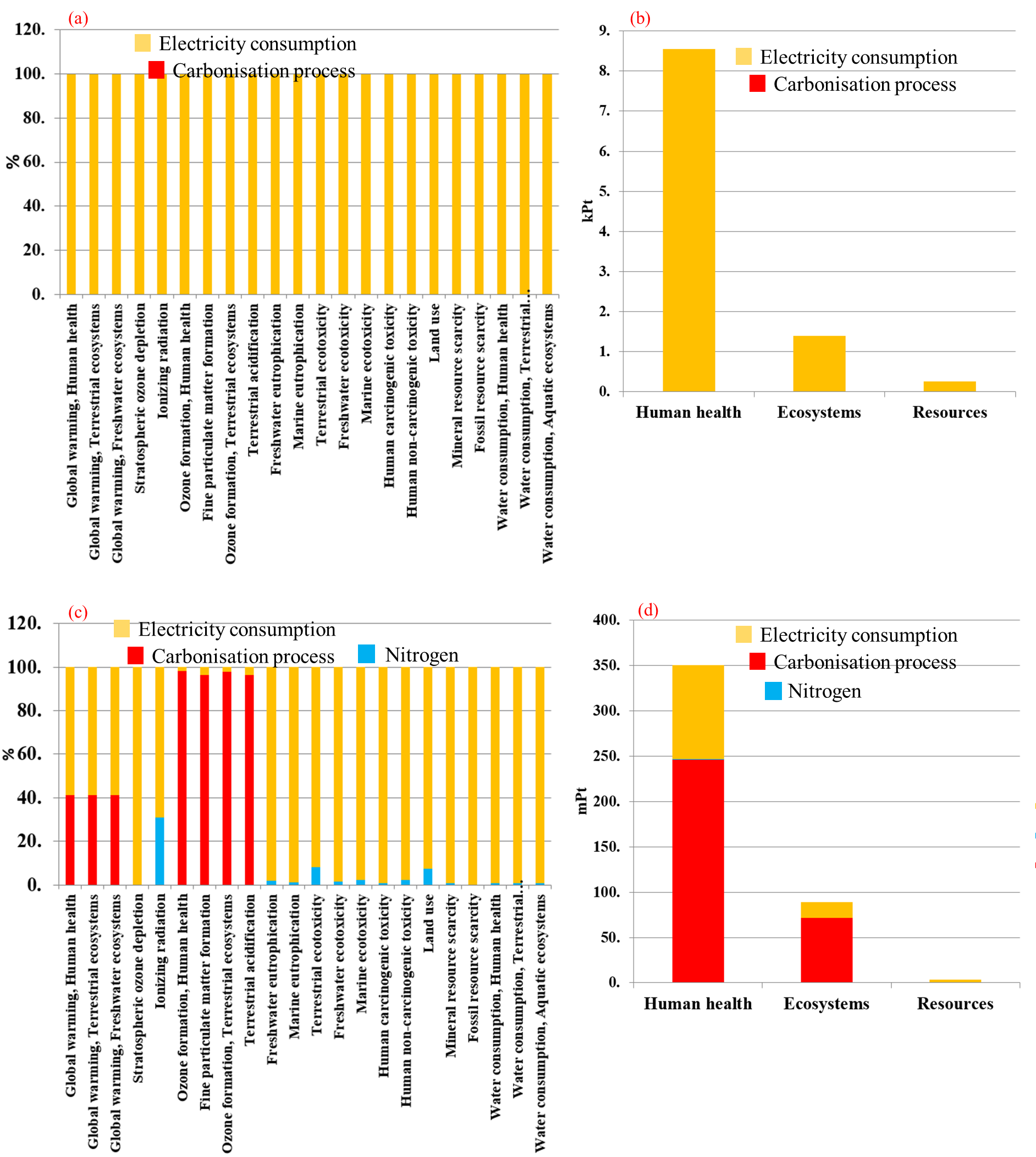


**Figure S22.** (a) Environmental impact profile of the carbonization process conducted at 1400 °C on a laboratory scale; (b) Damage assessment associated with the lab-scale carbonization process; (c) Environmental impact profile of the carbonization process at 1400 °C scaled to industrial levels; (d) Damage assessment of the large-scale carbonization process.


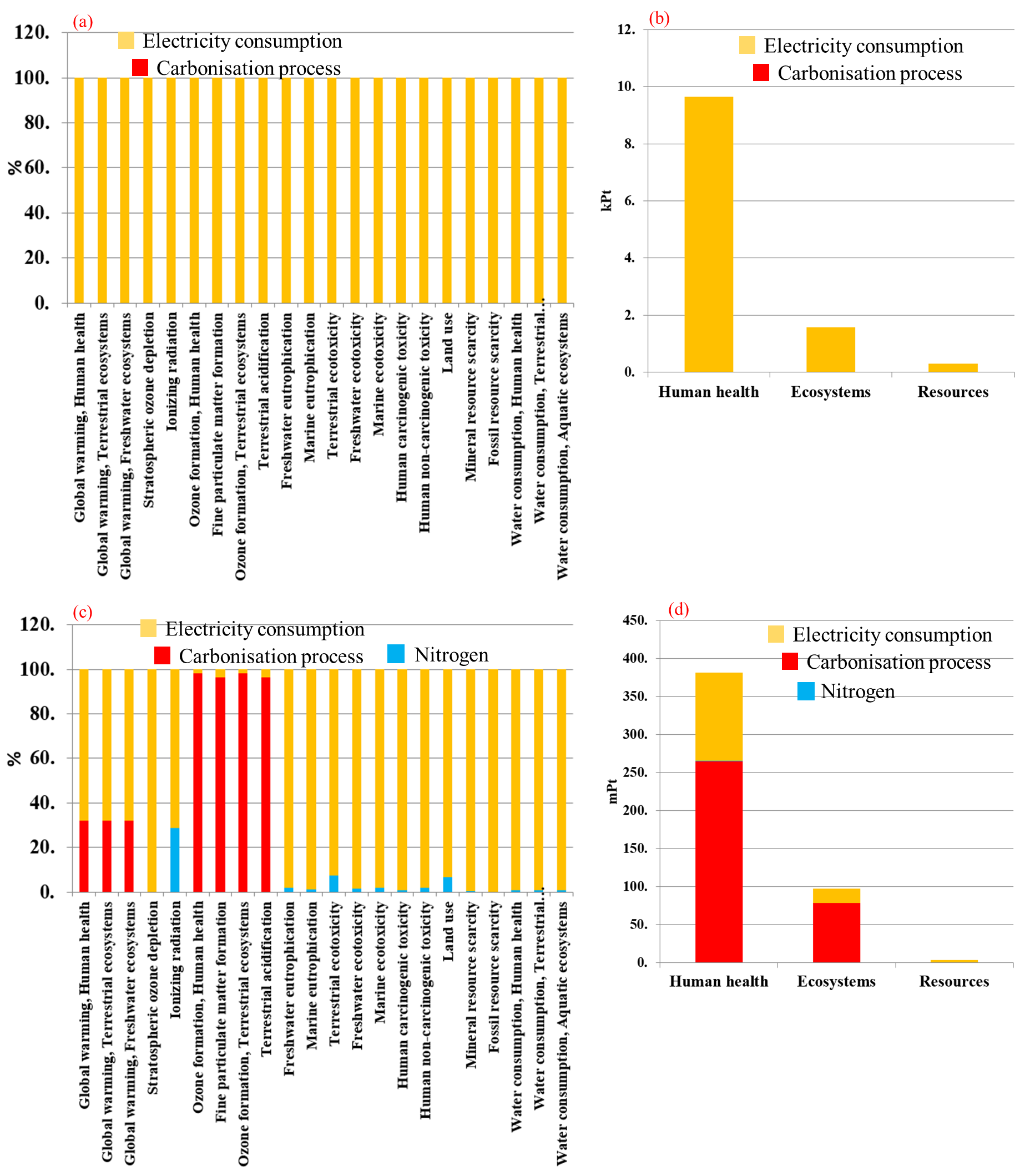


**Figure S23.** (a) Environmental impact profile of the carbonization process conducted at 1600 °C on a laboratory scale; (b) Damage assessment associated with the lab-scale carbonization process; (c) Environmental impact profile of the carbonization process at 1600 °C scaled to industrial levels; (d) Damage assessment of the large-scale carbonization process.

**
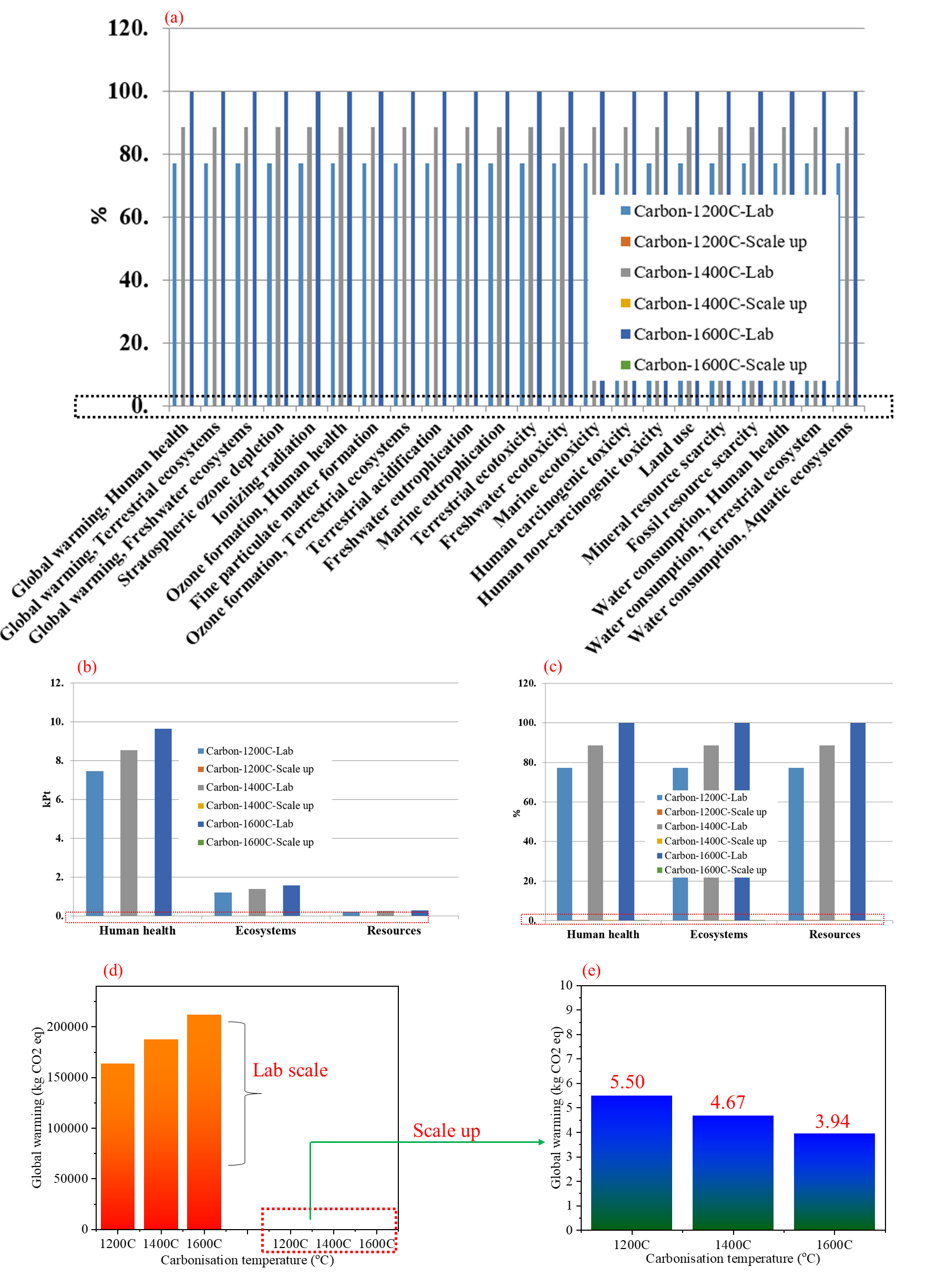
**

**Figure S24.** (a) Environmental impact profile of the carbonization process conducted on a laboratory scale and large scale; (b-c) Damage assessment associated with the carbonization process; (d) Head-to-head comparison of global warming potential (kg CO_2_ eq) of the carbonization process; (e) global warming potential (kg CO_2_ eq) of the carbonization process on large scale.

**Table S13.** Life Cycle Assessment (LCA) of the carbonization of waste CDs into carbon: Evaluating the environmental impact of the carbonization process at laboratory scale versus industrial scale-up.

| Impact category | Unit | Carbon-1600C-Scale up | Carbon-1600C-Lab | Carbon-1400C-Scale up | Carbon-1400C-Lab | Carbon-1200C-Scale up | Carbon-1200C-Lab |
| --- | --- | --- | --- | --- | --- | --- | --- |
| Global warming | kg CO2 eq | 3.943705586 | 211993.4917 | 4.675848243 | 187904.5572 | 5.506924233 | 163815.3699 |
| Stratospheric ozone depletion | kg CFC11 eq | 2.91083E-06 | 0.24247557 | 2.61986E-06 | 0.214921528 | 2.32889E-06 | 0.187367486 |
| Ionizing radiation | kBq Co-60 eq | -0.002054352 | -176.5057296 | -0.001842545 | -156.4482603 | -0.001630739 | -136.3907911 |
| Ozone formation, Human health | kg NOx eq | 0.746161787 | 1124.317825 | 0.646813462 | 996.5411155 | 0.580076271 | 868.7939028 |
| Fine particulate matter formation | kg PM2.5 eq | 0.00013478 | 11.10597096 | 0.000121453 | 9.843928806 | 0.000108126 | 8.581886651 |
| Ozone formation, Terrestrial ecosystems | kg NOx eq | 0.747197048 | 1210.565546 | 0.647745226 | 1072.987959 | 0.583724137 | 935.4426186 |
| Terrestrial acidification | kg SO2 eq | 0.273729248 | 830.3797978 | 0.2374531 | 736.0136617 | 0.211256953 | 641.6565255 |
| Freshwater eutrophication | kg P eq | 2.90086E-05 | 2.373656408 | 2.61603E-05 | 2.103922726 | 2.33119E-05 | 1.834189043 |
| Marine eutrophication | kg N eq | 3.6008E-06 | 0.296927097 | 3.24449E-06 | 0.263185381 | 2.88818E-06 | 0.229443666 |
| Terrestrial ecotoxicity | kg 1,4-DCB | 0.199111206 | 15561.45439 | 0.180437461 | 13793.1073 | 0.161763715 | 12024.76021 |
| Freshwater ecotoxicity | kg 1,4-DCB | 0.004983306 | 409.1925573 | 0.004492275 | 362.6934031 | 0.004001244 | 316.1942488 |
| Marine ecotoxicity | kg 1,4-DCB | 0.001479383 | 121.3329855 | 0.001333783 | 107.5451462 | 0.001188184 | 93.75730697 |
| Human carcinogenic toxicity | kg 1,4-DCB | 0.000433509 | 36.06317203 | 0.000390233 | 31.9650843 | 0.000346957 | 27.86699657 |
| Human non-carcinogenic toxicity | kg 1,4-DCB | 0.007291388 | 602.8889574 | 0.006567921 | 534.3788486 | 0.005844454 | 465.8687398 |
| Land use | m2a crop eq | 0.002746489 | 213.8040705 | 0.002489925 | 189.5081534 | 0.00223336 | 165.2122363 |
| Mineral resource scarcity | kg Cu eq | 0.001426716 | 118.0921068 | 0.001285006 | 104.6725492 | 0.001143295 | 91.25299163 |
| Fossil resource scarcity | kg oil eq | 1.391332606 | 115877.0652 | 1.252280128 | 102709.2168 | 1.11322765 | 89541.36853 |
| Water consumption | m3 | 0.000776554 | 63.94786457 | 0.000699816 | 56.68106178 | 0.000623079 | 49.41425898 |

**References**

[1] K. Wang *et al.*, "Low-Cost and High-Performance Hard Carbon Anode Materials for Sodium-Ion Batteries," *ACS Omega,* vol. 2, no. 4, pp. 1687-1695, 2017/04/30 2017, doi: 10.1021/acsomega.7b00259.

[2] Z. Guo *et al.*, "Investigating the Superior Performance of Hard Carbon Anodes in Sodium-Ion Compared With Lithium- and Potassium-Ion Batteries," *Advanced Materials,* vol. 35, no. 42, p. 2304091, 2023/10/01 2023, doi: <https://doi.org/10.1002/adma.202304091>.

[3] R. Fu *et al.*, "Surface oxo-functionalized hard carbon spheres enabled superior high-rate capability and long-cycle stability for Li-ion storage," *Electrochimica Acta,* vol. 260, pp. 430-438, 2018/01/10/ 2018, doi: <https://doi.org/10.1016/j.electacta.2017.12.043>.

[4] H. Moon *et al.*, "Assessing the Reactivity of Hard Carbon Anodes: Linking Material Properties with Electrochemical Response Upon Sodium- and Lithium-Ion Storage," *Batteries & Supercaps,* vol. 4, no. 6, pp. 960-977, 2021/06/01 2021, doi: <https://doi.org/10.1002/batt.202000322>.

[5] C. Matei Ghimbeu, A. Beda, and C. Vaulot, "Understanding the hard carbon's closed pore formation and Na-ion storage beyond the first charge/discharge cycle," *Carbon,* vol. 240, p. 120331, 2025/06/05/ 2025, doi: <https://doi.org/10.1016/j.carbon.2025.120331>.

[6] M. Sarkar, R. Hossain, J. Peng, N. Sharma, and V. Sahajwalla, "Electrochemical Compatibility of Microzonal Carbon in Ion Uptake and Molecular Insights into Interphase Evolution for Next‐Generation Li‐Ion Batteries," *Advanced Energy Materials,* vol. 14, no. 38, p. 2401977, 2024.
